# Supplementary material for: Yeast GPCR signaling reflects the fraction of occupied receptors, not the number
Source: Mol Syst Biol. 2016 Dec 29;12(12):898. doi: 10.15252/msb.20166910 (PMC5199120; doi:10.15252/msb.20166910)
Supplement: Supplementary file 1 — Appendix [file MSB-12-898-s001.pdf]

Appendix to Bush, Vasen et al. 2016

Alan Bush   Gustavo Vasen   Paula Dunayevich
Andreas Constantinou   Inés Lucía Patop   Matias Blaustein
Alejandro Colman-Lerner

Contents

1 Supplementary methods **1**
1.1 Plasmids and Primers 1
1.2 Yeast Strains 4
1.3 Cell treatments and microscopy methods 9
1.4 Statistical methods 10

2 Supplementary data **11**
2.1 Abundance of components of the pathway in different mediums 11

3 The carousel model of G protein activation **12**
3.1 Model definition 12
3.2 Symmetry assumptions: the simplified carousel model 15
3.3 Parameters of the simplified carousel model 17
3.4 Extended carousel model: Gβγ-Ste5 interaction 19
3.5 Extended carousel model: Receptor-RGS interaction 21
3.6 Restriction analysis of the simplified carousel model 24

1 Supplementary methods

1.1 Plasmids and Primers

Synthetic DNA fragments, primers and plasmids used in this study are listed in tables S1, S2 and S3, respectively.

Plasmid pRS406-hsRGS4-CFP was constructed using Gibson assembly [18] by cloning a synthesized gBlock DNA fragment (Integrated DNA Technologies; Table S1) coding for a yeast codon-optimized version of human *RGS4* (hsRGS4) with 60 bp 5' and 3' regions homologous to the *ACT1* promoter (pACT1) and CFP, respectively, into plasmid pRS406-CFP [12] linearized by digesting at the single *Eco*RI site between pACT1 and CFP.

Plasmid pRS406-K-hsRGS4-CFP was constructed by cloning a *Not*I/*Sac*I fragment carrying the kanMX6 resistance cassette from plasmid pFA6a-kanMX6 [30] into plasmid pRS406-hsRGS4-CFP downstream of the *ADH1* terminator.

hsRGS4

```
  1 gcttttagat ttttcacgct tactgctttt ttcttcccaa gatcgaaaat ttactgaatt
 61 aacagaattc ATGTGTAAGG GTTTGGCGGG TCTCCCAGCG TCTTGTTTGA GAAGTGCTAA
121 AGATATGAAG CACAGATTGG GTTTCTTGCT CCAAAAGAGT GATAGTTGTG AGCATAACTC
181 TTCTCACAAC AAGAAGGACA AGGTTGTTAT CTGTCAAAGA GTTTCGCAAG AAGAAGTAAA
241 GAAGTGGGCG GAAAGTTTGG AAAACTTGAT ATCGCACGAA TGCGGTTTGG CGGCGTTCAA
301 GGCGTTCCTC AAAAGTGAAT ACTCGGAAGA AAACATAGAC TTTTGGATAA GTTGTGAAGA
361 ATACAAGAAG ATAAAGTCTC CATCTAAACT CTCTCCAAAG GCGAAGAAGA TATACAACGA
421 GTTCATCTCT GTTCAAGCGA CTAAAGAAGT AAATCTCGAC TCTTGCACTA GGGAAGAAAC
481 TTCTCGTAAT ATGTTAGAAC CAACTATCAC TTGTTTTGAC GAAGCGCAAA AGAAGATATT
541 TAACTTGATG GAAAAGGACT CGTATAGAAG ATTTTTGAAG TCTCGTTTCT ACTTGGACTT
601 GGTAAATCCA TCTTCTTGTG GTGCTGAAAA GCAAAAGGGT GCTAAATCTT CTGCTGACTG
661 CGCTTCTCTC GTTCCACAAT GTggcggatc catgagtaaa ggagaagaac ttttcactgg
721 agttgtccca attcttgttg aattagatgg
```

STE2^{WT}

```
  1 aatgtgggcc acggctgcta ataatgcatc caaaacaacg ACAATTACTT CAGACTTTAC
 61 AACATCCACA GATAGGTTTT ATCCAGGCAC GCTGTCTAGC TTTCAAACTG ATAGTATCAA
121 CAACGATGCT AAAAGCAGTC TCAGAAGTAG ATTATATGAC CTATATCCTA GAAGGAAGGA
181 AACAACATCG GATAAACATT CGGAAAGAAC TTTTGTTTCT GAGACTGCAG ATGATATAGA
241 GAAAAATCAG TTTTATCAGT TGCCCACACC TACGAGTTCA AAAAATACTA GGATAGGACC
301 GTTTGCTGAT GCAAGTTACA AAGAGGGAGA AGTTGAACCC GTCGACATGT ACACTCCCGA
361 TACGGCAGCT GATGAGGAAG CCAGAAAGTT CTGGACTGAA GATAATAATA ATTTAcgtag
421 atccatgagt aaaggagaag aacttttcac tggag
```

STE2$^{20STA-7KR}$

```
  1 aatgtgggcc acggctgcta ataatgcatc caaaacaacg ACAATTACTT CAGACTTTAC
 61 AACATCCACA GATAGGTTTT ATCCAGGCAC GCTGTCTAGC TTTCAAACTG ATGCTATCAA
121 CAACGATGCT AGAGCCGCTC TCAGAGCTAG ATTATATGAC CTATATCCTA GAAGGAGGGA
181 AGCAGCAGCT GATAGACATG CTGAAAGAGC TTTTGTTGCT GAGGCTGCAG ATGATATAGA
241 GAGAAATCAG TTTTATCAGT TGCCCGCACC TGCTGCTGCA AGAAATGCTA GGATAGGACC
301 GTTTGCTGAT GCAGCTTACA GAGAGGGAGA AGTTGAACCC GTCGACATGT ACGCTCCCGA
361 TGCGGCAGCT GATGAGGAAG CCAGAAGGTT CTGGGCTGAA GATAATAATA ATTTAcgtag
421 atccatgagt aaaggagaag aacttttcac tggag
```

Table S1. Sequence of synthetic DNA used in this study. Lowercase indicate flanking sequences used for assembly.

To construct plasmid pMD45-K-hsRGS4-CFP we used PCR (primers 531 and 532) to amplify a DNA fragment from plasmid pRS406-K-hsRGS4-CFP which contained hsRGS4-CFP, the kanMX6 cassette [30], and 40 bp 5' and 3' regions homologous to the *STE2* ORF and terminator regions, respectively. Amplified DNA was then transformed for integration in a yeast strain in which the only copy of the *STE2* gene is a F204S mutant expressed from the centromeric plasmid pMD45 [14]. Transformants were selected on YPD agar plates supplemented with 350 µg/ml of antibiotic G418 (Geneticin; YPD-G418), after which modified plasmids were recovered by yeast plasmid rescue via transformation and amplification in *E. coli*, and verified by DNA sequencing.

Plasmid pBB13 was constructed by cloning a DNA fragment amplified by PCR from yeast genomic DNA (primers 223 and 224) containing the 403-916 nt region of the *STE2* ORF and flanked with *Kpn*I and *Rsr*II sites, respectively, into a pRS406 plasmid containing CFP and the *ADH1* terminator (tADH1). This resulted in the construct *Kpn*I-$STE2_{403-916}$-*Rsr*II-CFP-tADH1.

We constructed plasmids pRS406-STE2^{WT} and pRS406-STE2$^{20STA-7KR}$ using Gibson assembly [18] by cloning the corresponding synthetic DNA fragments (table S1) into plasmid pBB13 linearized by *Rsr*II digestion. These fragments contain the sequence coding for different variants of the C-terminal domain of Ste2p, flanked by 40 bp regions homologous to the plasmid DNA surrounding the *Rsr*II site. 7KR refers to mutations K337R, K352R, K358R, K374R, K387R, K400R and K422R [38]. 20STA refers to mutations S331A, S338A, S339A, S342A, T354A, T355A, S356A, S360A, T363A, S366A, T368A, T382A, T384A, S385A, S386A, T389A, S398A, T411A, T414A and T425A. This is similar to mutations described in [4], but we also included S356A and S342A. STE2$^{20STA-7KR}$ (also referred to as *STE2*^{RE} in the main text) contains all the previous mutations combined. All mutations were confirmed by DNA sequencing.

**Primer**	**Sequence**
223	gatctacggaccgTTGTTTTGGATGCATTATTAGCAGC
224	aaaagctgggtaccCAAGTCCTTCTTGTGGCTTC
416	gtttttcaaccatgtaaatttcctaattgggtaagtacaGAATTCGAGCTCGTTTAAAC
417	gatcataaaatagattgctcaatgaaggagccgcatcagaCATTTTGAGATCCGGGTTTT
418	gaaatcataatcccagctctgtctttacttctcatcgtccGAATTCGAGCTCGTTTAAAC
419	ttttggaagataattcatgcaacgtcctatttttatccacCATTTTGAGATCCGGGTTTT
468	gttataggttcaatttggtaattaaagatagagttgtaagCGGATCCCCGGGTTAATTAA
469	tcacacctccgcttaatttatacgtaatagtatttacagtGAATTCGAGCTCGTTTAAAC
502	ggaggtgttactgtcgtacgttcc
503	gcgctcacgttagtcacatctcagg
526	ggcgttataggttcaatttggtaattaaagatagagttgtaagATGTGTAAGGGTTTGGC
529	ctgtttgtgcaattgtacctgaagatgagtaagactctcaTGGATGGCGGCGTTAGTATC
530	gtttttctcaagacatgcttatctcttcgagtaatttaaatAAGTGGGCGGAAAGTTTGG
531	aggaagccagaaagttctggactgaagataataatatttaAAGTGGGCGGAAAGTTTGG
532	tataccgaaggtcacgaaattactttttcaaagccgtaaaTGGATGGCGGCGTTAGTATC

Table S2. DNA primers used in this study.

code	name	description	source
A1	pFA6a-kanMX6	P_{TEF}-kanMX6-T_{TEF}	[47]
A16	pFA6a-kanMX6-P_{GAL1}	P_{TEF}-kanMX6-T_{TEF}-P_{GAL1}	[30]
A42	pRS416	empty vector (CEN ARS4 URA3)	[37]
A47	pRS426	empty vector (2 µm URA3)	[10]
A168	pFA6a-hphMX4	P_{TEF}-hphMX4-T_{TEF}	[19]
A180	pRS406-CFP	pRS406-P_{ACT1}-CFP-T_{ADH1} (URA3)	[12]
A335	pFA6a-natMX6	P_{TEF}-natMX6-T_{TEF}	[24]
A382	pJBK008	P_{STE2}-STE2 (CEN1 ARS3 URA3)	[28]
A383	pMD45/pDJ452	P_{STE2}-STE2-F204S (CEN4 ARS1 URA3)	[14]
A392	pSZ111	P_{BMH2}-GAL4dbd-ER-VP16 in pRS405 (LEU2)	G. Pesce
A517	pRS406-hsRGS4-CFP	P_{ACT1}-hsRGS4-CFP-T_{ADH1} (URA3)	this work
A534	pBB13	pRS406-$STE2_{405-916}$-CFP-T_{ADH1} (URA3)	this work
A539	YEp24-STE2	P_{STE2}-STE2 (2 µm URA3)	[28]
A550	pRS406-K-hsRGS4-CFP	P_{ACT1}-hsRGS4-CFP-T_{ADH1}	this work
		⌞ P_{TEF}-kanMX6-T_{TEF} (URA3)	
A585	pMD45-K-hsRGS4-CFP	P_{STE2}-STE2-F204S-RGS(hsRGS4)-CFP	this work
		⌞ P_{TEF}-kanMX6-T_{TEF} (CEN4 ARS1 URA3)	
A587	pRS406-$STE2^{WT}$-CFP	pRS406-$STE2^{WT}_{402-1293}$-CFP-T_{ADH1} (URA3)	this work
A590	pRS406-$STE2^{20STA-7KR}$-CFP	pRS406-$STE2^{20STA-7KR}_{402-1293}$-CFP-$T_{ADH1}$ (URA3)	this work

Table S3. Plasmids used in this study.

1.2 Yeast Strains

All strains used in this work are derivatives of strain ACL379 (*MATa can1::HO-CAN1 ho::HO-ADE2 ura3-1 ade2-1 leu2-3 trp1-1 his3-11* Δ*bar1*) [12] of the W303a genetic background. Note that the *BAR1* gene coding for the α-factor specific protease has been deleted in this strain. Relevant genotypes of strains are listed in table S4.

Strains TCY394 [12], TCY3154 [12], and YPP3662 [46] have been previously described.

YFP-tagged genomic gene reporter strains TCY3071 (*FUS3*), TCY3072 (*STE7*), TCY3073 (*BMH1*), TCY3075 (*MSG5*), TCY3077 (*SST2*), and TCY3078 (*STE4*) were constructed by transforming strain ACL379 with PCR products amplified from plasmid pYFP-His3MX6 [12], containing the complete YFP coding sequence, the *ADH1* terminator, the His3MX6 cassette [30] (which codes for the *HIS3* homolog $his5^+$ gene from *S. pombe*), as well as 40 bp 5' and 3' in-frame sequence homology to the 3' end of the targeted gene for integration by homologous recombination.

We put *STE2* under control of the *GAL1* promoter (pGAL1) in strains YAB3930, YAB5302, and YAB5372 by transforming strains TCY3154, TCY394, and YPP3662, respectively, with a PCR product amplified from plasmid pFA6a-kanMX6-pGAL1 [30] (primers 416 and 417), carrying the KanMX6 resistance cassette, pGAL1, as well as 40 bp 5' and 3' sequence homology to the promoter region of *STE2*, such that integration replaced the 200 bp immediately upstream of the *STE2* ORF. We selected for transformants on YPD-G418 plates and screened for positive clones based on their ability to respond to α-factor in SC-Gal, but not in SC-Glu medium.

Similarly, we put *SST2* under control of pGAL1 in strain ACY5660 by transforming strain TCY394 with a PCR product amplified from plasmid pFA6a-kanMX6-pGAL1 [30] (primers 418 and 419) with 40 bp 5' and 3' sequence homology to the promoter region of *SST2*, such that integration replaced the 100 bp immediately upstream of the *SST2* ORF. We selected for transformants on YPGal plates (2% galactose) supplemented with 350 µg/ml G418 (YPGal-G418), and identified positive clones in halo assays as those forming normal halos on YPGal, but large halos on YPD.

To construct strain YAB5313 constitutively expressing hsRGS4 from the *ACT1* promoter (pACT1), we transformed strain YAB5302 with plasmid pRS406-hsRGS4-CFP digested with *Stu*I to target it for integration at the *URA3* locus. We selected for transformants on plates with synthetic medium lacking uracil (S –Ura) and identified positive clones by membrane associated CFP fluorescence.

To facilitate later PCR-mediated genomic modifications requiring selection with G418, we replaced the kanMX6 cassette in strains YAB5302 and YAB5372 by hphMX4 (which confers resistance to the antibiotic Hygromycin B), resulting in strains ACY5514 and ACY5588, respectively. To do this we digested plasmid pFA6a-hphMX4 [19] with *Bam*HI and *Sac*I and transformed it into said strains to promote homologous recombination between the shared *TEF* promoter (pTEF) and terminator (tTEF) sequences of hphMX4 and kanMX6 cassettes. We selected for transformants on YPD plates supplemented with 200 µg/ml of Hygromycin B (YPD-Hyg). Positive clones were then identified as those having gained resistance to hygromycin B to the detriment of G418 resistance, while retaining their ability to grow in medium lacking histidine (ACY5514) or supplemented with Nourseothricin (ACY5588), as the his3MX6 and natMX6 cassettes present in these strains also share homology with hphMX4 via pTEF and tTEF.

We constructed strains ACY5544, ACY5545, ACY5612, and ACY5613 expressing hsRGS4-CFP from the wild type *SST2* promoter by transformation of strains TCY394, ACY5514, YPP3662, and ACY5588, respectively, with a PCR product amplified from plasmid pRS406-K-hsRGS4-CFP using primers 526 and 529. This fragment contains the hsRGS4-CFP ORF and the kanMX6 resistance cassette flanked by regions of homology to the promoter and terminator regions of *SST2*, thus replacing the entire *SST2* ORF.

Likewise, we derived strains ACY5614 and ACY5615 expressing the *SST2*-hsRGS4-CFP chimera from strains YPP3662 and ACY5588, respectively, by transformation with a PCR product amplified from plasmid pRS406-K-hsRGS4-CFP using primers 526 and 530. This fragment contains the RGS domain of hsRGS4 (RGS$_{hsRGS4}$; AA 58–204) fused to CFP and the kanMX6 resistance cassette, flanked with 40 bp homology to the 3' end of the *SST2* DEP domain (DEP$_{SST2}$; AA 1–419) and to the *SST2* terminator, thus replacing the *SST2* RGS domain (RGS$_{SST2}$; AA 420–698).

Similarly, we constructed strain ACY5549 expressing RGS$_{hsRGS4}$ fused to *STE2* by transforming strain ACY5514 with a PCR product amplified from plasmid pRS406-K-

hsRGS4-CFP using primers primers 531 and 532. This fragment contains RGS_{hsRGS4}-CFP and the kanMX6 resistance cassette, flanked with 40 bp homology to the 3' end of the STE2 ORF and the *STE2* terminator, thus fusing RGS_{hsRGS4}-CFP to the STE2 ORF.

For all strains constructed with PCR products from the pRS406-K-hsRGS4-CFP plasmid, colonies were first selected on YPD-G418 plates and then restreaked onto S –His plates and/or YPD plates supplemented with the antibiotics the target strain was expected to be resistant to. This procedure avoided the selection of clones in which homologous recombination from the introduced PCR product had resulted in replacing a selection cassette already present in the genome, rather than modifying the target locus. Finally, all colonies were verified by expression of CFP fluorescence and by formation of small halos in the α-factor halo assay.

We deleted the *SST2* ORF in strains YAB5353 and ACY5609 by transforming strains YAB5313 and ACY5549, respectively, with a PCR product amplified from plasmid pFA6a-natMX6 [24] using primers 468 and 469. This DNA fragment contains the natMX6 resistance cassette flanked with 40 bp homology to the promoter and terminator regions of *SST2*, thus replacing the entire SST2 ORF. Transformants were selected on YPD plates supplemented with 100 µg/ml of the antibiotic Nourseothricin (YPD-Nat). We confirmed deletion of SST2 by colony PCR with primers 502 and 503. Note that we avoided passing through intermediate supersensitive strains with no functional RGS protein, as these tend to arrest spontaneously and accumulate sterile mutations.

We C-terminally tagged the genomic *STE2* gene with CFP in strains YGV5564 and YGV5627 by transforming strains YAB5302 and ACY5545, respectively, with plasmid pRS406-$STE2^{WT}$-CFP digested with *Cla*I. This modification also introduced the *ADH1* terminator (tADH1) after the *STE2*-CFP ORF. Similarly, we constructed strains YGV5563, YGV5626, YGV5630, and YGV5580 that express $STE2^{20STA-7KR}$-CFP (also referred to as $STE2^{RE}$ in the main text) from the genomic locus by transforming strains TCY394, ACY5544, ACY5545, and YAB5302, respectively, with plasmid pRS406-STE2-20STA-7KR-CFP digested with *Cla*I. These chimeric constructs were also followed by tADH1. Likewise, we made strain YAB3724 that expresses a CFP-tagged truncated from of *STE2* ($STE2^{1-305}$) by transforming strain ACL379 with plasmid pBB13 digested with *Cla*I. In all cases we selected for transformants on S –Ura plates and confirmed positive clones by expression of CFP fluorescence.

We introduced the GAL4-ER-VP16 (GEV) chimeric transcription factor [16] into strains YIP5370, YIP5581, ACY5620, and YGV5642 to control expression of *STE2* from the *GAL1* promoter by transforming strains YAB5353, YGV5564, ACY5609, and YGV5627, respectively, with plasmid pSZ111 digested with *Nde*I, targeting integration to the *BMH2* locus. pSZ111 was kindly provided by Dr. Gustavo Pesce and contains GEV under control of the *BMH2* promoter, which has low cell-to-cell variability (G. Pesce, personal communication). We selected for transformants in synthetic medium lacking leucine (S –Leu) and confirmed the clones phenotypically by their ability to respond to α-factor only in the presence of β-estradiol.

Expression of *STE2* variants from episomal single- or multicopy vectors in strains ACY5552 (empty; 2μ), ACY5553 (*STE2*; 2μ), YGV5666 (empty; CEN/ARS), YGV5667 (*STE2*; CEN/ARS), and YGV5668 (*STE2*-F204S; CEN/ARS) was achieved by transforming strain TCY394 with plasmids pRS426, YEp-STE2, pRS416, pJBK008, and pMD45, respectively. These plasmids were also transformed into strain ACY5544 expressing hsRGS4-

CFP in place of *SST2*, resulting in strains YGV5648 (empty; 2μ), YGV5649 (*STE2*; 2μ), YGV5669 (empty; CEN/ARS), YGV5670 (*STE2*; CEN/ARS), and YGV5671 (*STE2*-F204S; CEN/ARS), respectively. Furthermore, we transformed strain ACY5609 (expressing the *STE2*-RGS$_{hsRGS4}$ chimera from the *STE2* genomic locus) with plasmids pJBK008 and pMD45 to construct strains YGV5679 (*STE2*; CEN/ARS) and YGV5680 (*STE2*-F204S; CEN/ARS), respectively. Finally, we transformed strain ACY5660 with plasmid pMD45-K-hsRGS4-CFP to obtain strain ACY5662, which expresses the *STE2*-F204S-RGS$_{hsRGS4}$ chimera from a CEN/ARS episomal vector. In all cases selection was performed on S –Ura plates.

**Strain**	**[Parental]**	**Relevant Genotype**
ACL379	[W303a]	Parental strain of this study (1)
TCY394	[ACL379]	*prm1::*P_{PRM1}*-YFP::his5*$^+$ (1,2)
TCY3002	[ACL379]	*prm1::*P_{PRM1}*-YFP::his5*$^+$, Δ*ste2::TRP1* (1)
TCY3106	[ACL379]	Δ*ste5::natMX4* (1)
TCY3154	[ACL394]	*prm1::*P_{PRM1}*-YFP::his5*$^+$, *act1::*P_{ACT1}*-CFP::TRP1* (1,2)
TCY3071	[ACL379]	*fus3::FUS3-YFP::his5*$^+$
TCY3072	[ACL379]	*ste7::STE7-YFP::his5*$^+$
TCY3073	[ACL379]	*bmh2::BMH2-YFP::his5*$^+$
TCY3075	[ACL379]	*msg5::MSG5-YFP::his5*$^+$
TCY3077	[ACL379]	*sst2::SST2-YFP::his5*$^+$
TCY3078	[ACL379]	*ste4::STE4-YFP::his5*$^+$
YPP3661	[TCY3106]	Δ*ste5::natMX4*, *trp1::STE5-YFP*$\times$*3::TRP1* (3)
YPP3662	[TCY3106]	Δ*ste5::natMX4*, *trp1::STE5-YFP*$\times$*3::TRP1 (*$\times$*3)* (3,4)
YAB3724	[ACL379]	*ste2::*P_{STE2}*-STE2-T305-CFP::URA3*
YAB3930	[TCY3154]	*prm1::*P_{PRM1}*-YFP::his5*$^+$, *act1::*P_{ACT1}*-CFP::TRP1,*
	∟	*ste2::*P_{GAL1}*-STE2::kanMX6* (2)
YAB4075	[TCY3002]	*prm1::*P_{PRM1}*-YFP::his5*$^+$, Δ*ste2::TRP1* w/ pRS416 (1)
YAB4078	[TCY3002]	*prm1::*P_{PRM1}*-YFP::his5*$^+$, Δ*ste2::TRP1* w/ pJBK008 (1)
YAB4079	[TCY3002]	*prm1::*P_{PRM1}*-YFP::his5*$^+$, Δ*ste2::TRP1* w/ pMD45 (1)
YAB5302	[ACL394]	*prm1::*P_{PRM1}*-YFP::his5*$^+$, *STE2::*P_{GAL1}*-STE2::kanMX6* (2)
YAB5313	[YAB5302]	*prm1::*P_{PRM1}*-YFP::his5*$^+$, *STE2::*P_{GAL1}*-STE2::kanMX6,*
	∟	*ura3::*P_{ACT1}*-hsRGS4-CFP::URA3* (2)
YAB5353	[YAB5313]	*prm1::*P_{PRM1}*-YFP::his5*$^+$, *STE2::*P_{GAL1}*-STE2::kanMX6,*
	∟	*ura3::*P_{ACT1}*-hsRGS4-CFP::URA3*, Δ*sst2::natMX4,* (2)
YIP5370	[YAB5353]	*prm1::*P_{PRM1}*-YFP::his5*$^+$, *STE2::*P_{GAL1}*-STE2::kanMX6,*
	|	*ura3::*P_{ACT1}*-hsRGS4-CFP::URA3*, Δ*sst2::natMX4,*
	∟	*leu2::*P_{BMH2}*-GEV::LEU2* (2)
YAB5372	[YPP3662]	Δ*ste5::natMX4*, *trp1::STE5-YFP*$\times$*3::TRP1 (*$\times$*3),*
	∟	*STE2::*P_{GAL1}*-STE2::kanMX6* (4)
ACY5514	[YAB5302]	*prm1::*P_{PRM1}*-YFP::his5*$^+$, *STE2::*P_{GAL1}*-STE2::hphMX4* (2)

Strain	[Parental]	Relevant Genotype
ACY5544	[ACL394]	*prm1::*P_{PRM1}*-YFP::his5*$^{+}$*, sst2::*P_{SST2}*-hsRGS4-CFP::kanMX6* (2)
ACY5545	[ACY5514]	*prm1::*P_{PRM1}*-YFP::his5*$^{+}$*, STE2::*P_{GAL1}*-STE2::hphMX4*
	∟	*sst2::*P_{SST2}*-hsRGS4-CFP::kanMX6* (2)
ACY5549	[ACY5514]	*prm1::*P_{PRM1}*-YFP::his5*$^{+}$*, STE2::*P_{GAL1}*-STE2::hphMX4,*
	∟	*ste2::STE2-RGS(RGS4)-CFP::kanMX6* (2)
ACY5552	[ACL394]	*prm1::*P_{PRM1}*-YFP::his5*$^{+}$ w/ pRS426 (2)
ACY5553	[ACL394]	*prm1::*P_{PRM1}*-YFP::his5*$^{+}$ w/ YEp24-STE2 (2)
YGV5560	[ACL394]	*prm1::*P_{PRM1}*-YFP::his5*$^{+}$*, STE2::STE2-CFP::URA3* (2)
YGV5563	[ACL394]	*prm1::*P_{PRM1}*-YFP::his5*$^{+}$*, STE2::STE2-20STA-7KR-CFP::URA3* (2)
YGV5564	[YAB5302]	*prm1::*P_{PRM1}*-YFP::his5*$^{+}$*, STE2::*P_{GAL1}*-STE2::kanMX6*
	∟	*STE2::STE2-CFP::URA3* (2)
YGV5580	[YAB5302]	*prm1::*P_{PRM1}*-YFP::his5*$^{+}$*, STE2::*P_{GAL1}*-STE2::kanMX6,*
	∟	*STE2::STE2-20STA-7KR-CFP::URA3* (2)
YIP5581	[YGV5564]	*prm1::*P_{PRM1}*-YFP::his5*$^{+}$*, STE2::*P_{GAL1}*-STE2::kanMX6,*
	∟	*STE2::STE2-CFP::URA3, BMH2::*P_{BMH2}*-GEV::LEU2*(2)
ACY5588	[YAB5372]	Δ*ste5::natMX4, trp1::STE5-YFP*$\times$*3::TRP1 (*$\times$*3),*
	∟	*STE2::*P_{GAL1}*-STE2::hphMX4* (4)
ACY5609	[ACY5549]	*prm1::*P_{PRM1}*-YFP::his5*$^{+}$*, STE2::*P_{GAL1}*-STE2::hphMX4,*
	∟	*ste2::STE2-RGS(RGS4)-CFP::kanMX6,* Δ*sst2::natMX4* (2)
ACY5612	[YPP3662]	Δ*ste5::natMX4, trp1::STE5-YFP*$\times$*3::TRP1 (*$\times$*3),*
	∟	*sst2::*P_{SST2}*-hsRGS4-CFP::kanMX6* (4)
ACY5613	[ACY5588]	Δ*ste5::natMX4, trp1::STE5-YFP*$\times$*3::TRP1 (*$\times$*3),*
	∟	*STE2::*P_{GAL1}*-STE2::hphMX4, sst2::*P_{SST2}*-hsRGS4-CFP::kanMX6* (4)
ACY5614	[YPP3662]	Δ*ste5::natMX4, trp1::STE5-YFP*$\times$*3::TRP1 (*$\times$*3),*
	∟	*sst2::*P_{SST2}*-DEP(SST2)-RGS(hsRGS4)-CFP::kanMX6*(4)
ACY5615	[ACY5588]	Δ*ste5::natMX4, trp1::STE5-YFP*$\times$*3::TRP1 (*$\times$*3),*
	|	*STE2::*P_{GAL1}*-STE2::hphMX4,*
	∟	*sst2::*P_{SST2}*-DEP(SST2)-RGS(hsRGS4)-CFP::kanMX6* (4)
ACY5620	[ACY5609]	*prm1::*P_{PRM1}*-YFP::his5*$^{+}$*, STE2::*P_{GAL1}*-STE2::hphMX4,*
	|	*ste2::STE2-RGS(RGS4)-CFP::kanMX6,* Δ*sst2::natMX4,*
	∟	*BMH2::*P_{BMH2}*-GEV::LEU2* (2)
YGV5626	[ACY5544]	*prm1::*P_{PRM1}*-YFP::his5*$^{+}$*, sst2::*P_{SST2}*-hsRGS4-CFP::kanMX6,*
	∟	*STE2::STE2-20STA-7KR-CFP::URA3* (2)
YGV5627	[ACY5545]	*prm1::*P_{PRM1}*-YFP::his5*$^{+}$*, ste2::*P_{GAL1}*-STE2::hphMX4,*
	∟	*sst2::hsRGS4-CFP::kanMX6, STE2::STE2-CFP::URA3* (2)
YGV5630	[ACY5545]	*prm1::*P_{PRM1}*-YFP::his5*$^{+}$*, STE2::*P_{GAL1}*-STE2::hphMX4,*
	∟	*sst2::hsRGS4-CFP::kanMX6, STE2::STE2-20STA-7KR-CFP::URA3* (2)
YGV5642	[YGV5627]	*prm1::*P_{PRM1}*-YFP::his5*$^{+}$*, ste2::*P_{GAL1}*-STE2::hphMX4,*

Strain	[Parental]	Relevant Genotype
	│	*sst2::hsRGS4-CFP::kanMX6, STE2::STE2-CFP::URA3,*
	∟	*BMH2::*P_{BMH2}*-GEV::LEU2* (2)
YGV5648	[ACY5544]	*prm1::*P_{PRM1}*-YFP::his5*$^{+}$*, sst2::hsRGS4-CFP::kanMX6* w/ pRS426 (2)
ACY5652	[YGV5651]	*prm1::*P_{PRM1}*-YFP::his5*$^{+}$*, sst2::*P_{GAL1}*-SST2::kanMX6* w/ pRS426 (2)
ACY5653	[YGV5651]	*prm1::*P_{PRM1}*-YFP::his5*$^{+}$*, sst2::*P_{GAL1}*-SST2::kanMX6* w/ YEp24-STE2 (2)
YGV5649	[ACY5544]	*prm1::*P_{PRM1}*-YFP::his5*$^{+}$*, sst2::hsRGS4-CFP::kanMX6* w/ YEp24-STE2 (2)
ACY5660	[ACL394]	*prm1::*P_{PRM1}*-YFP::his5*$^{+}$*, sst2::*P_{GAL1}*-SST2::kanMX6* (2)
ACY5662	[ACY5660]	*prm1::*P_{PRM1}*-YFP::his5*$^{+}$*, sst2::*P_{GAL1}*-SST2::kanMX6*
	∟	w/ pMD45-K-hsRGS4-CFP (2)
YGV5666	[ACL394]	*prm1::*P_{PRM1}*-YFP::his5*$^{+}$ w/ pRS416 (2)
YGV5667	[ACL394]	*prm1::*P_{PRM1}*-YFP::his5*$^{+}$ w/ pJBK008 (2)
YGV5668	[ACL394]	*prm1::*P_{PRM1}*-YFP::his5*$^{+}$ w/ pMD45 (2)
YGV5669	[ACY5544]	*prm1::*P_{PRM1}*-YFP::his5*$^{+}$*, sst2::hsRGS4-CFP::kanMX6* w/ pRS416 (2)
YGV5670	[ACY5544]	*prm1::*P_{PRM1}*-YFP::his5*$^{+}$*, sst2::hsRGS4-CFP::kanMX6* w/ pJBK008 (2)
YGV5671	[ACY5544]	*prm1::*P_{PRM1}*-YFP::his5*$^{+}$*, sst2::hsRGS4-CFP::kanMX6* w/ pMD45 (2)
YGV5678	[ACY5609]	*prm1::*P_{PRM1}*-YFP::his5*$^{+}$*, STE2::*P_{GAL1}*-STE2::hphMX4,* (2)
	∟	*ste2::STE2-RGS(RGS4)-CFP::kanMX6,* Δ*sst2::natMX4* w/ pRS416
YGV5679	[ACY5609]	*prm1::*P_{PRM1}*-YFP::his5*$^{+}$*, STE2::*P_{GAL1}*-STE2::hphMX4,* (2)
	∟	*ste2::STE2-RGS(RGS4)-CFP::kanMX6,* Δ*sst2::natMX4* w/ pJBK008
YGV5680	[ACY5609]	*prm1::*P_{PRM1}*-YFP::his5*$^{+}$*, STE2::*P_{GAL1}*-STE2::hphMX4,* (2)
	∟	*ste2::STE2-RGS(RGS4)-CFP::kanMX6,* Δ*sst2::natMX4* w/ pMD45
ACL5682	[ACY5660]	*prm1::*P_{PRM1}*-YFP::his5*$^{+}$*, sst2::*P_{GAL1}*-SST2::kanMX6* w/ pJBK008 (2)
ACL5683	[ACY5660]	*prm1::*P_{PRM1}*-YFP::his5*$^{+}$*, sst2::*P_{GAL1}*-SST2::kanMX6* w/ pMD45 (2)

(1) Strains used in [12]. (2) Strains with the inhibitable allele *CDC28-as2* [12] (behaves as WT in the absence of inhibitor 1-NM-PP1). (3) Strains containing STE5-YFP×3 (STE5 fused in frame with three YFP in tandem), kindly provided by Peter Pryciak. (4) Strain with three genomic integrations of STE5-YFP×3.

Table S4. Relevant genotypes of yeast strains used in this study.

1.3 Cell treatments and microscopy methods

We prepared cells for microscopy as follows. We harvested exponential growing cells at an OD_{600} around 0.02 ($\approx 6 \times 10^5$ cells/ mL), briefly sonicated and placed them in a 384-well glass-bottom plate (BD Falcon, around 10^4 cells/well). To adhere the cells to the glass we pre-treated the wells with a solution of 1 mg/ml of concanavalin A type V (Sigma-Aldrich) for at least 20 minutes and washed twice with water. We prepared serial dilutions of chemically synthesized α-factor (Yale Small Scale Peptide Synthesis, New Haven, CT) in a factor 1 in $\sqrt{10} \approx 3.16$, or 1 in 3, using media supplemented with 40 µg/ml

of casein (Roche Applied Science) at pH 5.5, or 0.1% w/v PEG (MW 3550, Sigma), to block unspecific binding to plastic material [12]. For fluorescent proteins (FP) reporter dose-response (DoR) experiments we stimulated the cells at the indicated concentrations of α-factor with 10 µM of the inhibitor of Cdc28-as2 1-NM-PP1, incubated at 30°C for 2 hours, added cycloheximide to a final concentration of 100 µg/ml to inhibit translation and incubated at 30°C for at least 2 more hours to allow complete maturation of the FP.

When experiments involved induction of Ste2 by the GEV system, cells were incubated with different concentrations of β-estradiol for 3 hours at 30°C followed by 2 hours α-factor treatment in the corresponding β-estradiol dose. When using supersensitive strains like P_{GAL1}-SST2 in SC-Glucose (where SST2 expression is repressed), to minimize the ligand depletion that may occur at pM concentration, cells were diluted 30-fold ($OD_{600} \approx 0.0006$ or 1.8×10^4 cells/ mL), incubated with α-factor and then concentrated by centrifugation.

Fluorescence microscopy-based cytometry was done as described elsewhere [12, 20]. We acquired images using a fully motorized Olympus IX-81 inverted microscope, equipped with LED illumination (CoolLED), Zero Drift autofocus system (Olympus), CoolSnapHQ2 cooled CCD camera (Photometrix), adequate filter cubes (Chroma Technologies Corp. 41028 for YFP and Hilyte488, and 31044v2 for CFP) and an Olympus 60X PlanApo oil immersion objective (NA 1.4). We used our software Cell-ID [12] for image segmentation and our R package Rcell [8] for analysis and visualization of the dataset. Determination of total cell, membrane associated and membrane recruitment of fluorescence was done essentially as detailed elsewhere [8, 20].

We used fluorescent-α-factor tagged with HiLyte488 (kindly provided by D.G. Drubin [42]) to measure receptor abundance over time. We incubated cells with 0 or 50 nM of unlabeled α-factor for 2 hours and then treated with inhibitor medium (SC medium containing 10 mM fluoride (NaF or KF) and 10 mM azide (NaN_3)) for 30 min. Then, we washed cells by centrifugation 4 times in inhibitor medium to dissociate unlabeled α-factor, in order to reveal Ste2 binding sites by fluorescent-α-factor. Given the slow unbinding rate of unlabeled pheromone [27], we performed controls to verify that this protocol effectively removed the unlabeled α-factor. Due to the slow binding dynamics of the fluorescent pheromone (Figure EV1C and [46]), we incubated for at least 3 hours at 30°C to reach binding equilibrium. To correct for the autofluorescence of dead cells in the fluorescent-pheromone channel, we subtracted the signal from cells treated identically but without addition of labeled-*alpha*-factor.

We did halo assays in the following manner: 100 µL of exponential cell cultures (OD_{600}=0.1 or $\approx 3 \times 10^5$ cells) were spread on agar plates of adequate medium. A sterile Whatman filter paper disk was placed on the surface of the agar and 5 µL of α-factor (0.1 nmol, 170 ng) was then placed on the disk. Plates were incubated at 30°C for 2-4 days before imaging.

1.4 Statistical methods

In Figures 2G, 6A and 6B, to account for day-to-day differences in α-factor preparations, we normalized pheromone concentration as follows. For each strain and experiment, we fitted the data to a Hill function and then divided the α-factor concentrations by the correspondent EC_{50}. The resulting DoR were therefore centered at 1. In order to recapture the sensitivity of each strain, we re-scaled the α-factor concentration using the average EC50 of each strain among the different experiments.

2 Supplementary data

2.1 Abundance of components of the pathway in different mediums

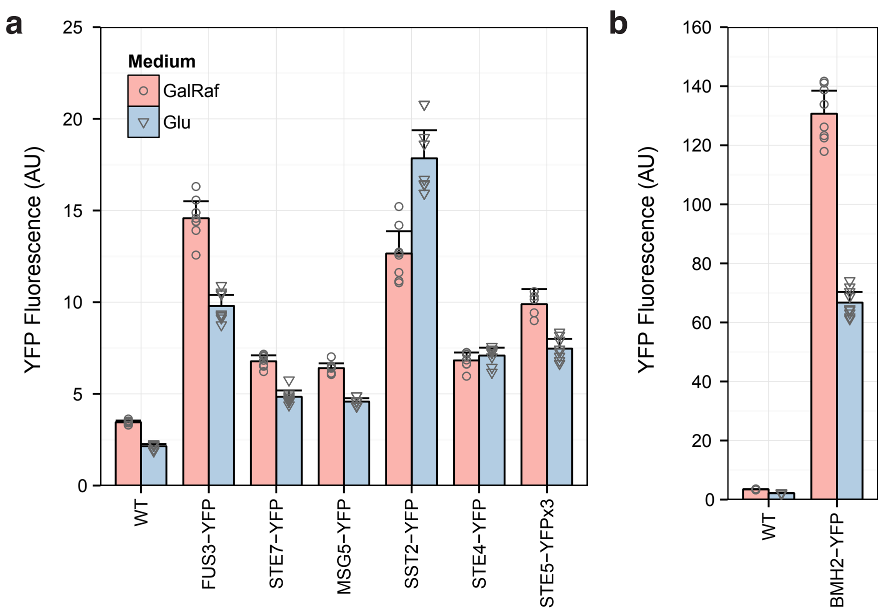

Figure S1. **a**) Fluorescence of strains with different components of the pheromone pathway tagged with YFP, grown in SC-Glu (blue bars, triangles) or SC-GalRaf (red bars, circles). **b**) Same measurements for Bmh2-YFP, a protein not related to the pheromone pathway. In both cases WT strain ACL379 was included as a autofluorescence control. Note the different y-scales.

As shown in Figure EV1B, the level of Ste2^{GPCR} is greatly reduced in the WT strain grown in medium with Galactose and Raffinose (SC-GalRaf), reaching levels 70% lower than the same strain grown in medium with Glucose (SC-Glu). Because of this unexpected result, we wondered if the abundance of other components of the pathway was also affected by the carbon source of the medium.

To this end, we tagged with YFP the endogenous copy of different components of the pheromone response pathway. Due to the low abundance of Ste5 [39], we used a strain in which *STE5* was tagged with 3 YFP in tandem to increase the fluorescence signal. We grew these strains in either SC-Glu or SC-GalRaf overnight, and acquired epifluorescence images as described in the methods section.

Although there are significant differences between the abundances of some of the components of the pathway when grown in SC-Glu or SC-GalRaf (Table S5), the relative difference is much less than in the case of Ste2^{GPCR}. Interestingly Bmh2, a protein not related to the pheromone pathway, shows a sugar-dependent change in its abundance much greater than the observed effect for components of the pathway. It is also noteworthy that Sst2^{RGS} shows the strongest YFP signal among the pathway components, suggesting in particular that it is more abundant than Ste4$^{\mathrm{G}\beta}$.

		YFP fluorescence – WT (AU)			
**Strain**	**Genotype**	GalRaf$^{(1)}$	Glu$^{(1)}$	GalRaf/Glu$^{(2)}$	**p-value**$^{(3)}$
TCY3071	FUS3-YFP	11 ± 1	7.6 ± 0.8	1.45 ± 0.21	0.00037
TCY3072	STE7-YFP	3.3 ± 0.4	2.7 ± 0.4	1.23 ± 0.23	0.011
TCY3075	MSG5-YFP	3.0 ± 0.3	2.4 ± 0.2	1.22 ± 0.17	0.0016
TCY3077	SST2-YFP	9 ± 1	16 ± 2	0.59 ± 0.12	0.00037
TCY3078	STE4-YFP	3.4 ± 0.5	4.9 ± 0.5	0.68 ± 0.12	0.00031
YPP3661	STE5-YFP×3	6.4 ± 0.7	5.3 ± 0.6	1.21 ± 0.19	0.016
TCY3073	BMH2-YFP	127 ± 9	65 ± 5	1.97 ± 0.20	0.000093

(1) Mean ± Standard deviation of the average YFP fluorescence signal per well, corrected by the autofluorescence of ACL379 (WT strain) in the indicated mediums. (2) SC-GalRaf to SC-Glu YFP fluorescence ratio ± propagated uncertainty. (3) p-value calculated by a permutation test (*perm* package for R).

Table S5. Corrected fluorescence values, ratios and significance for the data shown in Figure S1.

3 The carousel model of G protein activation

3.1 Model definition

The carousel model of G-protein activation results from the combination of the ternary complex model [13] and the G-protein activation cycle (Figures 1D and 1E of main text). The resulting model has many species and parameters, but can be conveniently represented in a 3D scheme as shown in Figure 3A of the main text, that evidences the symmetries in the model.

The axial reactions (violet arrows in Figure 3A), represent binding and unbinding of the ligand L with the receptor R. The *on*-rate of these reactions are given by the $k_{on}^{\square \cdot \square}$ parameters, were the square superscripts denote the binding partners. For example $k_{on}^{L \cdot R}$ is the *on*-rate for free receptor with the ligand (not explicitly represented in the scheme), while $k_{on}^{L \cdot RG}$ [1] is the *on*-rate for the G-protein coupled receptor with the ligand. The *off*-rates for the ligand-receptor complexes are written in the same notation. For example, $k_{off}^{L \cdot RGt}$ is the *off*-rate of ligand from the receptor coupled to $G\alpha^{GTP}$ [2].

The radial reactions (blue arrows in Figure 3A) represent the coupling between the different states of the Gα subunit and the receptor. These reactions are also expressed in terms of *on*-rates and *off*-rates using the same notation as before. For example, the coupling between free receptor and Gα^{GDP} is given by $k_{on}^{R \cdot Gd}$.

[1] In the model's parameters G denotes the heterotrimeric G-protein $G_{\alpha\beta\gamma}^{GDP}$, Gt represents Gα^{GTP} and Gd stands for Gα^{GDP}.

[2] In the model's variable names, the GTP and GDP superscripts are abbreviated to T and D respectively.

$$\frac{d(R)}{dt} = -\left(k_{on}^{L\cdot R}\,L\,R - k_{off}^{L\cdot R}\,LR\right) - \left(k_{on}^{R\cdot G}\,R\,G - k_{off}^{R\cdot G}\,RG\right) - \left(k_{on}^{R\cdot Gt}\,R\,Gt - k_{off}^{R\cdot Gt}\,RGt\right) - \left(k_{on}^{R\cdot Gd}\,R\,Gd - k_{off}^{R\cdot Gd}\,RGd\right) \tag{1}$$

$$\frac{d(G)}{dt} = -\left(k_{Ef}^{G}\,G\right) + \left(k_{Af}^{Gd}\,Gd\,G\beta\gamma - k_{Ar}^{Gd}\,G\right) - \left(k_{on}^{R\cdot G}\,R\,G - k_{off}^{R\cdot G}\,RG\right) - \left(k_{on}^{LR\cdot G}\,LR\,G - k_{off}^{LR\cdot G}\,LRG\right) \tag{2}$$

$$\frac{d(Gt)}{dt} = +\left(k_{Ef}^{G}\,G\right) - \left(k_{Hf}^{Gt}\,Gt\right) + \left(k_{Ef}^{Gd}\,Gd\right) - \left(k_{on}^{R\cdot Gt}\,R\,Gt - k_{off}^{R\cdot Gt}\,RGt\right) - \left(k_{on}^{LR\cdot Gt}\,LR\,Gt - k_{off}^{LR\cdot Gt}\,LRGt\right) \tag{3}$$

$$\frac{d(RG)}{dt} = +\left(k_{on}^{R\cdot G}\,R\,G - k_{off}^{R\cdot G}\,RG\right) - \left(k_{Ef}^{RG}\,RG\right) + \left(k_{Af}^{RGd}\,RGd\,G\beta\gamma - k_{Ar}^{RGd}\,RG\right) - \left(k_{on}^{L\cdot RG}\,L\,RG - k_{off}^{L\cdot RG}\,LRG\right) \tag{4}$$

$$\frac{d(RGt)}{dt} = +\left(k_{Ef}^{RG}\,RG\right) - \left(k_{Hf}^{RGt}\,RGt\right) + \left(k_{Ef}^{RGd}\,RGd\right) + \left(k_{on}^{R\cdot Gt}\,R\,Gt - k_{off}^{R\cdot Gt}\,RGt\right) - \left(k_{on}^{L\cdot RGt}\,L\,RGt - k_{off}^{L\cdot RGt}\,LRGt\right) \tag{5}$$

$$\frac{d(RGd)}{dt} = +\left(k_{Hf}^{RGt}\,RGt\right) - \left(k_{Ef}^{RGd}\,RGd\right) - \left(k_{Af}^{RGd}\,RGd\,G\beta\gamma - k_{Ar}^{RGd}\,RG\right) - \left(k_{on}^{L\cdot RGd}\,L\,RGd - k_{off}^{L\cdot RGd}\,LRGd\right) + \left(k_{on}^{R\cdot Gd}\,R\,Gd - k_{off}^{R\cdot Gd}\,RGd\right) \tag{6}$$

$$\frac{d(LR)}{dt} = +\left(k_{on}^{L\cdot R}\,L\,R - k_{off}^{L\cdot R}\,LR\right) - \left(k_{on}^{LR\cdot G}\,LR\,G - k_{off}^{LR\cdot G}\,LRG\right) - \left(k_{on}^{LR\cdot Gt}\,LR\,Gt - k_{off}^{LR\cdot Gt}\,LRGt\right) - \left(k_{on}^{LR\cdot Gd}\,LR\,Gd - k_{off}^{LR\cdot Gd}\,LRGd\right) \tag{7}$$

$$\frac{d(Gd)}{dt} = -\left(k_{Ef}^{Gd}\,Gd\right) + \left(k_{Hf}^{Gt}\,Gt\right) - \left(k_{Af}^{Gd}\,Gd\,G\beta\gamma - k_{Ar}^{Gd}\,G\right) - \left(k_{on}^{R\cdot Gd}\,R\,Gd - k_{off}^{R\cdot Gd}\,RGd\right) - \left(k_{on}^{LR\cdot Gd}\,LR\,Gd - k_{off}^{LR\cdot Gd}\,LRGd\right) \tag{8}$$

$$\frac{d(G\beta\gamma)}{dt} = +\left(k_{Ef}^{G}\,G\right) + \left(k_{Ef}^{RG}\,RG\right) + \left(k_{Ef}^{LRG}\,LRG\right) - \left(k_{Af}^{Gd}\,Gd\,G\beta\gamma - k_{Ar}^{Gd}\,G\right) - \left(k_{Af}^{RGd}\,RGd\,G\beta\gamma - k_{Ar}^{RGd}\,RG\right) - \left(k_{Af}^{LRGd}\,LRGd\,G\beta\gamma - k_{Ar}^{LRGd}\,LRG\right) \tag{9}$$

$$\frac{d(LRG)}{dt} = +\left(k_{on}^{LR\cdot G}\,LR\,G - k_{off}^{LR\cdot G}\,LRG\right) - \left(k_{Ef}^{LRG}\,LRG\right) + \left(k_{Af}^{LRGd}\,LRGd\,G\beta\gamma - k_{Ar}^{LRGd}\,LRG\right) + \left(k_{on}^{L\cdot RG}\,L\,RG - k_{off}^{L\cdot RG}\,LRG\right) \tag{10}$$

$$\frac{d(LRGt)}{dt} = +\left(k_{Ef}^{LRG}\,LRG\right) - \left(k_{Hf}^{LRGt}\,LRGt\right) + \left(k_{Ef}^{LRGd}\,LRGd\right) + \left(k_{on}^{LR\cdot Gt}\,LR\,Gt - k_{off}^{LR\cdot Gt}\,LRGt\right) + \left(k_{on}^{L\cdot RGt}\,L\,RGt - k_{off}^{L\cdot RGt}\,LRGt\right) \tag{11}$$

$$\frac{d(LRGd)}{dt} = +\left(k_{Hf}^{LRGt}\,LRGt\right) - \left(k_{Ef}^{LRGd}\,LRGd\right) - \left(k_{Af}^{LRGd}\,LRGd\,G\beta\gamma - k_{Ar}^{LRGd}\,LRG\right) + \left(k_{on}^{L\cdot RGd}\,L\,RGd - k_{off}^{L\cdot RGd}\,LRGd\right) + \left(k_{on}^{LR\cdot Gd}\,LR\,Gd - k_{off}^{LR\cdot Gd}\,LRGd\right) \tag{12}$$

The angular reactions represent progression through the G-protein activation cycle. The green arrows represent the exchange of GDP for GTP in the Gα subunit. The rates for these reactions are given by the $k_{Ef}^{\square}$ (Exchange forward) parameters, where the square

superscript indicates the molecular complex the Gα subunit is part of. Note that any Gα bound to GDP can undergo the exchange reaction, which includes the heterotrimeric G$\alpha\beta\gamma$ protein and dissociated Gα^{GDP}, either uncoupled from receptor, coupled to free receptor or coupled to ligand-occupied receptor.

Nucleotide exchange is not a simple reaction but the result of several elemental reactions; release of GDP from Gα^{GDP}, binding of GTP to the free binding site of Gα, transition of Gα^{GTP} to a conformation with low affinity for G$\beta\gamma$, and dissociation of G$\beta\gamma$ from Gα^{GTP}. Of these reactions, the dissociation of GDP from Gα has been reported to be the rate-limiting step in some systems [26, 32]. All reactions are in principle reversible, so a reverse exchange were GTP is replaced by GDP is theoretically possible. We decided to neglect this reverse reaction as it is very unfavorable given the large [GTP]/[GDP] ratio in metabolically active cells [43]. Therefore, we assume that guanine nucleotide exchange in Gα is adequately modeled by a irreversible reaction.

The red arrows in Figure 3A represent the hydrolysis of the gamma phosphate of GTP bound to Gα. This results in the conversion of Gα^{GTP} to Gα^{GDP} and inorganic phosphate. The rates of these reactions are given by $k_{Hf}^{\square}$ (Hydrolysis forward), where the square superscript indicates the complex Gα^{GTP} is part of. For example, k_{Hf}^{LRGt} is the hydrolysis rate of Gα^{GTP} coupled to ligand occupied receptor. We assume this reaction to be irreversible due to the large amount of free energy released during GTP hydrolysis. Note that in this model we assume that the concentrations of GTP, GDP and inorganic phosphate are not significantly influenced by the G-protein activation cycle and remain constant, therefore they are implicitly considered in the kinetic parameters.

The orange arrows in the carousel scheme represent the association between G$\beta\gamma$ and Gα^{GDP}. These reactions are modeled as reversible associations with rates $k_{Af}^{\square}$ and $k_{Ar}^{\square}$ (Association forward and reverse), where the superindexes indicate the partners Gα^{GDP} is bound to. For example k_{Af}^{RGd} is the rate for the association between G$\beta\gamma$ and Gα^{GDP} coupled to unoccupied receptor.

With these kinetic rates and following the laws of mass action, we defined a system of ordinary differential equations (ODEs) that describes the evolution of every specie in the model (Equations 1 to 12). For ease of reading, we grouped in parenthesis the forward and reverse terms of reversible reactions, and separated in lines terms that correspond to different reactions.

From these equations, we can see there are some conservations in the model, namely, the total amounts of R, Gα and G$\beta\gamma$ are conserved (equations 13 to 15). These conservations reduce the dimensionality of the ODE system from 12 to 9. The ligand concentration L is assumed to be constant, and equal to the applied ligand (*i.e.* no ligand depletion).

$$R_{tot} = R + RG + RGt + RGd + LR + LRG + LRGt + LRGd \tag{13}$$

$$G\alpha_{tot} = G + Gt + Gd + RG + RGt + RGd + LRG + LRGt + LRGd \tag{14}$$

$$G\beta\gamma_{tot} = G + RG + LRG + G\beta\gamma \tag{15}$$

The complete carousel model has 38 parameters. Taking into account thermodynamic micro-reversibility considerations, we can impose a relation between parameters for every independent close loop of reversible reactions [51]. These conditions ensure that if there are two reversible routes from one specie to an other, both have the same equilibrium constant or, in other words, that there is no net tendency for the molecules to *circulate*

in a preferential direction. Taking into account these relationships reduces the number of parameters to 33 and makes the model sounder and more biologically realistic.

Using a notation inspired by the Cubic Ternary Complex model [49], we encoded the micro-reversibility considerations using adimensional parameters (denoted by Greek letters) that relate the equilibrium constants for different reactions (equations 16 to 28) .

$$K_d^{L\cdot R} = \frac{k_{off}^{L\cdot R}}{k_{on}^{L\cdot R}} \quad (16)$$

$$K_d^{L\cdot RG} = \frac{k_{off}^{L\cdot RG}}{k_{on}^{L\cdot RG}} = \lambda\, K_d^{L\cdot R} \quad (17)$$

$$K_d^{L\cdot RGt} = \frac{k_{off}^{L\cdot RGt}}{k_{on}^{L\cdot RGt}} = \lambda_t\, K_d^{L\cdot R} \quad (18)$$

$$K_d^{L\cdot RGd} = \frac{k_{off}^{L\cdot RGd}}{k_{on}^{L\cdot RGd}} = \lambda_d\, K_d^{L\cdot R} \quad (19)$$

$$K_d^{R\cdot G} = \frac{k_{off}^{R\cdot G}}{k_{on}^{R\cdot G}} \quad (20)$$

$$K_d^{R\cdot Gt} = \frac{k_{off}^{R\cdot Gt}}{k_{on}^{R\cdot Gt}} = \eta\, K_d^{R\cdot G} \quad (21)$$

$$K_d^{R\cdot Gd} = \frac{k_{off}^{R\cdot Gd}}{k_{on}^{R\cdot Gd}} = \rho\, K_d^{R\cdot G} \quad (22)$$

$$K_d^{LR\cdot G} = \frac{k_{off}^{LR\cdot G}}{k_{on}^{LR\cdot G}} = \lambda\, K_d^{R\cdot G} \quad (23)$$

$$K_d^{LR\cdot Gt} = \frac{k_{off}^{LR\cdot Gt}}{k_{on}^{LR\cdot Gt}} = \lambda_t\, \eta\, K_d^{R\cdot G} \quad (24)$$

$$K_d^{LR\cdot Gd} = \frac{k_{off}^{LR\cdot Gd}}{k_{on}^{LR\cdot Gd}} = \lambda_d\, \rho\, K_d^{R\cdot G} \quad (25)$$

$$K_d^{Gd\cdot G\beta\gamma} = \frac{k_{Ar}^{Gd}}{k_{Af}^{Gd}} \quad (26)$$

$$K_d^{RGd\cdot G\beta\gamma} = \frac{k_{Ar}^{RGd}}{k_{Af}^{RGd}} = \frac{1}{\rho}\, K_d^{Gd\cdot G\beta\gamma} \quad (27)$$

$$K_d^{LRGd\cdot G\beta\gamma} = \frac{k_{Ar}^{LRGd}}{k_{Af}^{LRGd}} = \frac{\lambda}{\lambda_d\, \rho}\, K_d^{Gd\cdot G\beta\gamma} \quad (28)$$

3.2 Symmetry assumptions: the simplified carousel model

As a staring point, and to simplify the analysis, we decided to study a simplified version of the carousel model, in which me make several symmetry assumptions:

1) Binding of ligand to receptor is independent on whether the receptor is coupled or not to Gα, and to the state of this subunit (Gα^{GTP}, Gα^{GDP} or G$\alpha\beta\gamma$).

$$\lambda = \lambda_t = \lambda_d = 1 \quad (29)$$

$$k_{off}^{L\cdot R} = k_{off}^{L\cdot RG} = k_{off}^{L\cdot RGt} = k_{off}^{L\cdot RGd} \quad (30)$$

Note that equation 29 defines equal equilibrium constants, while equation 30 defines equal kinetic rates.

This assumption is supported by experiments in yeast, in which deletion of *GPA1*$^{G\alpha}$ shows very small changes in affinity, from $K_d^{\alpha\mathrm{F}\cdot STE2} = 3.4\,\mathrm{nM}$ in WT cells to $4.8\,\mathrm{nM}$ in Δ*gpa1*$^{G\alpha}$ cells [3]. On the other hand, experiments with membrane preparations do show a effect on $K_d^{\alpha\mathrm{F}\cdot Ste2}$, depending on the presence of GTP-γ-S (supposed to lock $G\alpha$ in the active state), but only in non-physiological conditions (high ionic strength and pH) [6].

In other GPCR systems, there is strong evidence for an effect of G-protein coupling to the receptor on the receptor-ligand affinity. In fact, this was the original motivation

for the Ternary Complex Model [13]. We decided to do the current assumption because it appears to be the case in the yeast pheromone pathway [3], and because it greatly simplifies the analysis of the model.

2) Coupling between Gα and the receptor does not depend on the state of Gα.

$$\eta = \rho = 1 \tag{31}$$

$$k_{off}^{R\cdot G} = k_{off}^{R\cdot Gt} = k_{off}^{R\cdot Gd} = k_{off}^{LR\cdot G} = k_{off}^{LR\cdot Gt} = k_{off}^{LR\cdot Gd} \tag{32}$$

Inmuno-precipitation experiments show that somewhat less Gpa1$^{G\alpha}$ co-precipitates with Ste2^{GPCR} after treatment with saturating α-factor than with no pheromone [50]. This suggests some degree of reduction in affinity between the receptor and Gα when the pathway is active, and therefore more molecules are in the Gα^{GTP} state. Nevertheless, this loss of affinity is partial and hard to estimate from the available data. The co-precipitation experiment gives no information on the kinetic rates of these interactions. In order to reduce the number of parameters of the model, we made the assumption described in equations 31 and 32, even though these might not be exact in the case of the pheromone pathway.

3) Association between Gα^{GDP} and G$\beta\gamma$ does not depend on whether Gα is coupled or not to the receptor, or the state of the receptor.

$$\rho = 1 \quad \text{and} \quad \lambda = \lambda_d \tag{33}$$

$$k_{Ar}^{Gd} = k_{Ar}^{RGd} = k_{Ar}^{LRGd} \tag{34}$$

Note that the assumption for the equilibrium constants (equation 33) is implied by the previous two assumptions (equations 29 and 31) and the micro-reversibility conditions.

4) The exchange rate of GDP for GTP on Gα^{GDP} is not affected by G$\beta\gamma$ binding.

$$k_{Ef}^{G} = k_{Ef}^{Gd}, \quad k_{Ef}^{RG} = k_{Ef}^{RGd} \quad \text{and} \quad k_{Ef}^{LRG} = k_{Ef}^{LRGd} \tag{35}$$

Dissociation of GDP from rabbit $Gs\alpha$ and $Go\alpha$ (the rate limiting step in the exchange reaction), is slower in the presence of G$\beta\gamma$ [7], but this effect disappears as Mg^{2+} concentrations increase [26]. Given the [Mg^{2+}] of yeast cytoplasm [21], this suggests that G$\beta\gamma$ should have only a small effect on GDP exchange in physiological conditions (see discussion in [41] and YeastPheromoneModel.org).

5) The hydrolysis rate of GTP by Gα^{GTP} coupled to receptor, does not depend on whether the receptor is bound to ligand or not.

$$k_{Hf}^{RGt} = k_{Hf}^{LRGt} \tag{36}$$

Note that this does not imply that the rate of GTP hydrolysis by free (receptor uncoupled) Gα^{GTP} should be the same as for receptor-coupled Gα^{GTP} (see below). To our knowledge there is no mechanism that could explain a change in GTP hydrolysis rate due to the ligand-occupation state of the receptor.

6) There are equimolar amounts of Gα and G$\beta\gamma$.

$$G_{tot} \equiv G\alpha_{tot} = G\beta\gamma_{tot} \tag{37}$$

Measurements of pathway components abundance by quantitative western blot resulted in an estimation of 2390 ± 262 molecules cell^{-1} for Gpa1$^{G\alpha}$ and 2045 ± 107 molecules cell^{-1} for Ste4$^{\text{G}\beta}$ [39], which are consistent with equimolar amounts of these components.

These assumptions have different degrees of experimental support for the yeast pheromone pathway. Regardless if they hold exactly for this system, we believe they constitute a reasonable simplification of the carousel model that facilitates the analysis, while allowing for interesting behaviors. Further relaxations of these assumptions could result in new and interesting features, but their analysis goes beyond the scope of this work.

3.3 Parameters of the simplified carousel model

These symmetry assumptions result in a simplified carousel model, with 13 free parameters to determine. Seven of these parameters have been determined for the yeast's pheromone response. These are the abundances of the components of the system, the *on* and *off* rates of α-factor with the receptor, the basal exchange rate of GDP for GTP in Gα^{GDP}, the basal hydrolysis rate of GTP in Gα^{GTP} and the increase in this rate induced by Sst2^{RGS} (see Table 1 in main text).

Other parameters have been measured in other GPCR signaling systems and can be considered as a good estimation of the correspondent parameters in the pheromone response pathway. These are the coupling and uncoupling rate of GPCRs with G proteins [1,2,23], and the exchange rate of GDP for GTP in Gα^{GDP} stimulated by the GEF activity of occupied receptors [5,32]. A thorough review of the evidence for these rates can be found in Ty Thomson's PhD thesis [41] and in the YeastPheromoneModel.org [40] site.

There are three parameters for which (to our knowledge) there are no experimental estimations available in any GPCR system. We estimated the values of these parameters as follows.

We estimated $K_d^{Gd \cdot G\beta\gamma}$ based on our experimental measurement of the affinity between Ste5 and G$\beta\gamma$ [8]. To do this, we first considered the closed cycle of reversible reactions between Gα^{GDP}, G$\beta\gamma$ and Ste5, shown in equation 38.

$$\begin{array}{ccc} G_\alpha^D + G_{\beta\gamma} + \text{Ste5} & \overset{K_1}{\rightleftharpoons} & G_\alpha^D + G_{\beta\gamma}\text{Ste5} \\ K_3 \downarrow\uparrow & & K_2 \downarrow\uparrow \\ G_{\alpha\beta\gamma}^D + \text{Ste5} & \overset{K_4}{\rightleftharpoons} & G_{\alpha\beta\gamma}^D\text{Ste5} \end{array} \tag{38}$$

Here, K_1 to K_4 represent the correspondent association constants. Because of micro-reversibility considerations [51], these constants have to satisfy the following equation.

$$K_2 = \frac{K_3}{K_1} K_4 \tag{39}$$

K_1 is the reciprocal of the dissociation constant between Ste5 and free G$\beta\gamma$, a parameter for which we have experimentally determined an upper bound of $\frac{1}{K_1} = K_d^{G\beta\gamma\cdot\mathrm{Ste5}} < 0.65\,\mathrm{nM}$ in a previous work [8]. K_3 is the reciprocal of $K_d^{Gd\cdot G\beta\gamma}$, the value we attempt to estimate. We expect K_3 to be relatively large, because Gα^{GDP} tightly binds G$\beta\gamma$ [11]. On the other hand, Ste5 cannot bind the heterotrimeric G protein (only free G$\beta\gamma$), therefore we assume $K_4 \approx 0$. In consequence, as follows from equation 39, the affinity constant between Gα^{GDP} and the complex between G$\beta\gamma$ and Ste5 is negligible ($K_2 \approx 0$). In other words, Ste5 and Gα^{GDP} bind to G$\beta\gamma$ in a mutually exclusive manner. In unstimulated cells, where almost all Gα is in the GDP form, Gα^{GDP} has to outcompete Ste5 for G$\beta\gamma$. Otherwise the pathway would be active in the absence of pheromone. This implies that $K_3 \gg K_1$, or equivalently that $K_d^{Gd\cdot G\beta\gamma} \ll K_d^{G\beta\gamma\cdot\mathrm{Ste5}} < 0.65\,\mathrm{nM}$. On the other hand, the strongest protein-protein interactions reported in heterodimeric complexes have dissociation constants in the pM range [9]. Therefore, we decided to use a estimated value of $K_d^{Gd\cdot G\beta\gamma} = 0.01\,\mathrm{nM}$, which is much lower (higher affinity) than our upper bound for the Ste5 and G$\beta\gamma$ dissociation constant, but well within the physiological range.

We chose the association rate k_{Af}^{Gd} such that the hydrolysis of GTP, and not the association of $G\alpha$ with $G\beta\gamma$, is the limiting step in the deactivation of G-protein.

Finally, we set the value of the exchange rate for G protein coupled to unoccupied receptor to the same value as for uncoupled G protein ($k_{Ef}^{RG} = k_{Ef}^{G}$).

All the components of the carousel model are permanently associated to the plasma membrane. The ODEs that define the model (Equations 1 to 12) are expressed in terms of concentrations of the different species, therefore we need to define a reaction volume. We considered a *perimembrane* space spanning $\Delta h = 50\,\mathrm{nm}$ across the plasma membrane, that contains all the components of the model. We defined the perimembrane volume as $V_{perimembrane} = 4\pi R_{cell}^2 \Delta h = 3.92\,\mathrm{fl}$, where $R_{cell} = 2.5\,\mathrm{\mu m}$. ODE models of reaction networks assume well-mixed compartments, an assumption unlikely to hold due to the relatively slow diffusion of membrane associated components and the geometry of the perimembrane volume. Another complication arises from the fact that at high doses of α-factor the signaling proteins concentrate at the shmoo tip, in a much smaller volume, within the first hour of stimulation (see Figure S4 in [46]). We decided to assume a well-mixed compartment of constant size as a starting point in our work, as this greatly simplifies the analysis of the model. The qualitative conclusions obtained do not depend strongly on the reaction volume used in the model, therefore one can interpret the model as the interactions that take place in a membrane *microdomain*, which can be considered a well-mixed compartment.

Calibrated western-blot estimations of the abundance of Ste2^{GPCR} receptors result in 6622 ± 361 molecules cell^{-1} [39]. Ste2^{GPCR} receptors have been shown to form dimmers *in vivo* [33], therefore we estimated around 3300 dimmers cell^{-1}. Although we assumed monomeric receptors in our model, we used the abundance of dimmers to estimate R_{tot}. The rationale for this is that the model mainly focuses in the coupling between receptors and G proteins, and we believe that the number of *coupling partners* is better estimated

in this manner. As described in the main text, the model shows robustness to receptor abundance, so the exact number used for R_{tot} has no influence on the conclusions of this work.

The regulator of G-protein signaling (RGS) is not explicitly represented in the carousel model. Nevertheless, its effect can be captured by the parameters of the model, specifically in the GTP hydrolysis rates. In the pheromone response pathway of yeast, Sst2^{RGS} physically interacts with the cytoplasmic C-termial domain of Ste2^{GPCR}, and this interaction is required for Sst2's activity on $G\alpha$ [4]. The effect and localization of the RGS can be captured in the parameters of the model, if the GTP hydrolysis rate is much greater for $G\alpha$ coupled to receptor than for uncoupled $G\alpha$ (equation 40). Note that this equation is satisfied by the parameter in Table 1 in main text, and symmetry assumption 5 (equation 36).

$$k_{Hf}^{LRGt} \approx k_{Hf}^{RGt} >> k_{Hf}^{Gt} \tag{40}$$

3.4 Extended carousel model: Gβγ-Ste5 interaction

To asses the effect of downstream components on the dynamic of the G-protein cycle, we extended the carousel model to include the interaction between free G$\beta\gamma$ and the scaffold protein Ste5. This interaction mediates the recruitment of Ste5 to the membrane and is required for signal transmission in the yeast pheromone system [34].

$$G\beta\gamma + \mathrm{Ste5} \underset{k_{off}^{G\beta\gamma\cdot\mathrm{Ste5}}}{\overset{k_{on}^{G\beta\gamma\cdot\mathrm{Ste5}}}{\rightleftharpoons}} G\beta\gamma\mathrm{Ste5} \tag{41}$$

This extension adds four new parameter to the model; the total amount of scaffold protein (Ste5$_{tot}$), the *off*-rate between G$\beta\gamma$ and Ste5 ($k_{off}^{G\beta\gamma\cdot\mathrm{Ste5}}$), the dissociation constant for this interaction ($K_d^{G\beta\gamma\cdot\mathrm{Ste5}} = k_{off}^{G\beta\gamma\cdot\mathrm{Ste5}}/k_{on}^{G\beta\gamma\cdot\mathrm{Ste5}}$), and the cytoplasmic volume (V_{cyt}). Note that, as discussed in section 3.3, we are assuming that Ste5 and Gα bind in a mutually exclusive manner to G$\beta\gamma$.

- Like most components of the pathway, the abundance of Ste5 has been measured by quantitative western blot, resulting in Ste5$_{tot}$ $= 480 \pm 60$ molecules/cell [39]. Note that this is one of the components in lowest abundance.

- We estimated $k_{off}^{G\beta\gamma\cdot\mathrm{Ste5}}$ based on a FRAP experiment [45]: cells expressing Ste5-GFP were stimulated with α-factor 3 µM for 90 minutes. In these conditions Ste5 localized to the shmoo tip, where it was photobleached. The fluorescence at the shmoo tip recovered in a half time of $\tau_{1/2} = 8.2 \pm 1.3$ s [45]. Because cytoplasmic diffusion is much faster than the binding reaction, this recovery time is mainly determined by the *off*-rate between Ste5 and G$\beta\gamma$, which we can estimate as $k_{off}^{G\beta\gamma\cdot\mathrm{Ste5}} = \frac{log(2)}{\tau_{1/2}} = 0.085 \pm 0.013\ s^{-1}$.

- In a previous work we have experimentally measured the affinity between Ste5 and G$\beta\gamma$ [8]. We found that the affinity is modulated during the response, starting with a high "peak" affinity (low dissociation constant) of $K_{d,peak}^{G\beta\gamma\cdot\mathrm{Ste5}} < 0.65nM$, which then goes to a "steady-state" value of $K_{d,ss}^{G\beta\gamma\cdot\mathrm{Ste5}} = 17 \pm 9nM$, within the first five minutes of the response. This modulation of the affinity results in a peak and decline

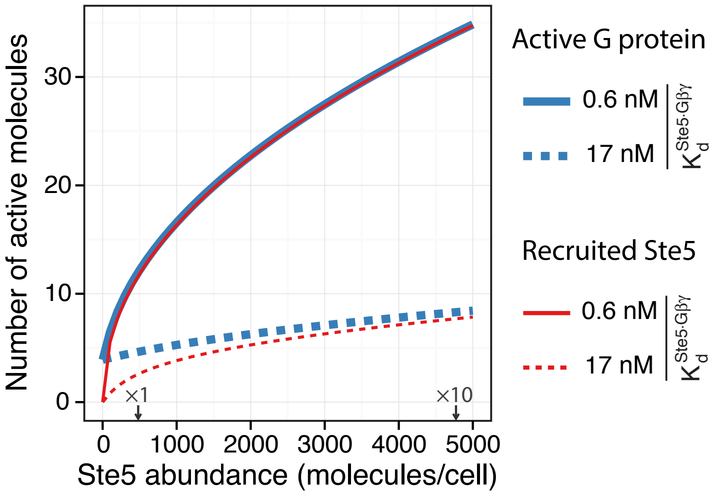

Figure S2. Simulated basal activation *vs.* Ste5 abundance. The blue curves represent the number of $G\beta\gamma$ subunits dissociated from $G\alpha$ and the red curves the number of membrane recruited Ste5 molecules. The solid curves were simulated using the peak affinity $K_{d,peak}^{G\beta\gamma\cdot \text{Ste5}} = 0.6nM$, while the dashed curves used the steady-state affinity $K_{d,ss}^{G\beta\gamma\cdot \text{Ste5}} = 17nM$. The estimated Ste5 abundances for *wt* strains (x1) and for a strain with a tenfold increase (x10) are indicated on the x axis.

dynamics for Ste5's membrane recruitment. Therefore, we used two values for this parameter in the following simulations; $K_{d,peak}^{G\beta\gamma\cdot \text{Ste5}} = 0.6nM$ and $K_{d,ss}^{G\beta\gamma\cdot \text{Ste5}} = 17nM$.

- As Ste5 resides in the cytoplasm before it is recruited to the membrane, we had to defined the volume of the cytoplasmic compartment V_{cyt}. Note that the relevant volume is the one accessible to Ste5. We estimated this volume to be 36.4 fl based on the soft X-ray tomography data presented in [44].

We wanted to study the extent to which Ste5 can sequester $G\beta\gamma$ form $G\alpha$ in an unstimulated system, based on our estimation of the model's parameters. To do this we simulated the basal levels of active G protein (*i.e.* $G\beta\gamma$ dissociated from $G\alpha$, either free or bound to Ste5) and membrane recruited Ste5, for different total abundances of Ste5.

As can be observed in Figure S2 there are around 4 active G proteins (0.2% of the total 2,042 G proteins simulated) in the absence of Ste5. When the simulation was run using the steady-state affinity $K_{d,ss}^{G\beta\gamma\cdot \text{Ste5}} = 17nM$, we found a very small increase in the number of active G proteins, reaching 8 molecules (0.4%) for a tenfold over-expression of Ste5. On the other hand, when using the peak affinity $K_{d,peak}^{G\beta\gamma\cdot \text{Ste5}} = 0.6nM$, the effect is somewhat stronger; 12 G-protein molecules (0.6%) are active with *wt* abundance of Ste5 and 34 G-proteins (1.7%) are activated with a tenfold increase in the amount of Ste5.

For steady-state simulations as the ones used in this work, the most reasonable affinity value to use is $K_{d,ss}^{G\beta\gamma\cdot \text{Ste5}} = 17nM$. In any case, based on the value used for the model's parameters, the sequestration effect of Ste5 on $G\beta\gamma$ appears to affect a very small percentage of the total G-protein. Therefore, for the rest of this work we neglected the effect of Ste5 on the activation of the G protein.

We do not have the quantitative data required to do a similar analysis with other binding partners of $G\beta\gamma$, like Far1 or Ste20. We therefore make the reasonable assumption that they also have a negligible effect on the activation of the G protein, as the model suggest for Ste5. It is interesting to note that there is four times more G protein than Ste5 molecules [39]. Therefore Ste5 cannot titrate free $G\beta\gamma$ at high pheromone doses, leaving sites available for the interaction with other partners as Far1.

3.5 Extended carousel model: Receptor-RGS interaction

Due to the importance of the receptor-RGS interaction suggested by our previous results, we decided to explicitly include the RGS component in our model. To express all the possible states of the receptor in a convenient way, we introduce the following notation,

$$\underset{z}{xRy} \quad \text{where} \begin{cases} x \in \{\emptyset, L\} \\ y \in \{\emptyset, G, Gd, Gt\} \\ z \in \{\emptyset, \textit{RGS}\} \end{cases} \tag{42}$$

where x can be either nothing or L, y can be nothing, G, Gd or Gt (representing G$\alpha\beta\gamma$, Gα^{GDP} or Gα^{GTP}, respectively) and z can be nothing or *RGS*. Therefore, this extended model has $2 \times 4 \times 2 = 16$ species representing different states of the receptor, 4 species for the free G protein (G$\alpha\beta\gamma$, Gα^{GDP}, Gα^{GTP} and G$\beta\gamma$) and one specie representing free cytoplasmic RGS.

As can be seen, adding explicitly the RGS protein and its interaction with the receptor greatly increases the complexity of the model, going from 12 to 21 species, from 35 to 81 reactions and from 38 to 85 total parameters (Figure EV3A). This is an example of combinatorial explosion in the complexity of a model.

In order to keep the model tractable, we did additional symmetry or independence assumptions (the numeration is correlative with the assumptions in section 3.2).

7) Binding affinity and kinetics between ligand and receptor is not influenced by the binding of the RGS to the receptor.

$$K_d^{L\cdot Ry} = K_d^{\underset{\mathsf{RGS}}{L\cdot Ry}} \quad \text{for} \quad y \in \{\emptyset, G, Gd, Gt\} \tag{43}$$

$$k_{off}^{L\cdot Ry} = k_{off}^{\underset{\mathsf{RGS}}{L\cdot Ry}} \quad \text{for} \quad y \in \{\emptyset, G, Gd, Gt\} \tag{44}$$

Several studies show that *STE2*^{GPCR} alleles with no C-terminal domain have the same affinity for α-factor than *wt* receptors [15, 28, 35]. As Sst2^{RGS} binds to the C-terminal domain of Ste2^{GPCR}, this suggests there is no effect of the RGS on ligand binding affinity. Note that combining this assumption with symmetry assumption 1 results in the same affinity and kinetics of ligand binding for all receptor species (*i.e.* all violet "axial" arrows in Figure EV3A).

8) Coupling between Gα and the receptor is not influenced by the receptor-RGS interaction.

$$K_d^{xR\cdot y} = K_d^{\underset{\mathsf{RGS}}{xR\cdot y}} \quad \text{for} \quad y \in \{G, Gd, Gt\}, x \in \{\emptyset, L\} \tag{45}$$

$$k_{off}^{xR\cdot y} = k_{off}^{\underset{\mathsf{RGS}}{xR\cdot y}} \quad \text{for} \quad y \in \{G, Gd, Gt\}, x \in \{\emptyset, L\} \tag{46}$$

Two studies propose that Sst2^{RGS} increases the affinity between Ste2^{GPCR} and Gpa1$^{G\alpha}$ [29, 36]. In the first study the authors found that the pheromone induced

cell-cycle arrest is robust to receptor abundance in *wt* cells, but not in $\Delta sst2^{\mathrm{RGS}}$ cells [36]. The second study shows that $\mathrm{Sst2}^{\mathrm{RGS}}$ is required for the dominant-negative effect of loss-of-function mutations in $STE2^{\mathrm{GPCR}}$ [29]. Their interpretation of these observations was that the receptor pre-couples with the G protein (forming a stable complex), and that $\mathrm{Sst2}^{\mathrm{RGS}}$ is required for this interaction. However, Weiner et al. specifically looked for, but were unable to find any evidence that $\mathrm{Sst2}^{\mathrm{RGS}}$ modulates the affinity between receptor and G protein [48]. In this work we propose an alternative explanation for the mentioned observations, namely, that unoccupied receptors inactivate G proteins due to their interaction with $\mathrm{Sst2}^{\mathrm{RGS}}$. In this view it is not necessary to postulate any modulation of the receptor-G protein affinity due to $\mathrm{Sst2}^{\mathrm{RGS}}$.

This assumption combined with assumptions 2 results in the same rates for coupling between $\mathrm{G}\alpha$ (in any state) to the receptor (ligand-occupied or not). In other words, all blue "radial" arrows in Figure EV3A are equivalent.

9) Association between the receptor-$\mathrm{G}\alpha^{\mathrm{GDP}}$ complex and $\mathrm{G}\beta\gamma$, does not depend on whether the RGS is bound to the receptor or not.

$$K_d^{xRGd\cdot G\beta\gamma} = K_d^{\overset{xRGd\cdot G\beta\gamma}{\mathsf{RGS}}} \quad \text{for} \quad x \in \{\emptyset, L\} \tag{47}$$

$$k_{off}^{xRGd\cdot G\beta\gamma} = k_{off}^{\overset{xRGd\cdot G\beta\gamma}{\mathsf{RGS}}} \quad \text{for} \quad x \in \{\emptyset, L\} \tag{48}$$

To our knowledge there is no evidence for a change in the rate of re-association of the G protein due to the interaction between the $\mathrm{Sst2}^{\mathrm{RGS}}$ and $\mathrm{Ste2}^{\mathrm{GPCR}}$. Taken together with symmetry assumption 3, this implies that all orange "angular" reactions in Figure EV3A are equivalent.

10) The receptor induced exchange rate of GDP for GTP in $\mathrm{G}\alpha$ is not influenced by binding of the RGS to the receptor.

$$k_{Ef}^{xRGd} = k_{Ef}^{\overset{xRGd}{\mathsf{RGS}}} \quad \text{for} \quad x \in \{\emptyset, L\} \tag{49}$$

This assumption indicates that the correspondent green "angular" arrows in the left and right carousel schemes of Figure EV3A are equivalent.

11) The interaction between the receptor and the RGS is not influenced by the sate of the receptor.

$$K_d^{xRy\cdot \mathsf{RGS}} = K_d^{R\cdot \mathsf{RGS}} \quad \text{for all} \quad x \in \{\emptyset, L\} \text{ and } y \in \{\emptyset, G, Gd, Gt\} \tag{50}$$

$$k_{off}^{xRy\cdot \mathsf{RGS}} = k_{off}^{R\cdot \mathsf{RGS}} \quad \text{for all} \quad x \in \{\emptyset, L\} \text{ and } y \in \{\emptyset, G, Gd, Gt\} \tag{51}$$

In the work by Ballon et al. where the DEP domains of $\mathrm{Sst2}^{\mathrm{RGS}}$ were first described, the authors showed that the affinity between $\mathrm{Sst2}^{\mathrm{RGS}}$ and $\mathrm{Ste2}^{\mathrm{GPCR}}$ is

reduced when the C-terminal domain of the receptor is phosphorylated by the kinases Yck1 and Yck2 [4]. Because phosphorylation of the receptor by Yck1/2 is promoted by pheromone binding to the receptor [25], this suggests that the affinity of $\mathrm{Sst2^{RGS}}$ for pheromone bound receptors should be lower than for unoccupied receptors ($K_d^{LRy\cdot\mathsf{RGS}} > K_d^{Ry\cdot\mathsf{RGS}}$ for $y \in \{\emptyset, G, Gd, Gt\}$). This can have interesting consequences, as ligand-occupied receptors could have reduced GAP activity besides their increased GEF activity, therefore enhancing the functional antagonism with unoccupied receptors. Furthermore, this could explain different behaviors at different receptor occupancies that correspond to high and low pheromone doses.

Despite this, as we are not modeling the receptor's phosphorylation and turnover explicitly, and in order to keep the extended carousel model as simple as possible, we decided to do the current symmetry assumption.

In terms of the scheme of Figure EV3A this means that the horizontal black arrows for all receptor species are equivalent.

The fundamental difference between RGS bound receptors and receptors with no RGS is in the GTP hydrolysis rates of the Gα subunits coupled to them. The GAP activity, and therefore the enhanced hydrolysis rate, is only present on the complexes with RGS (compare red "angular" arrows in Figure EV3A). This condition is mathematically defined by equation 52.

$$k_{Hf}^{Gt} \approx k_{Hf}^{xRGt} \ll k_{Hf}^{\underset{\mathsf{RGS}}{xRGt}} \quad \text{for} \quad x \in \{\emptyset, L\} \tag{52}$$

Taking into account the additional symmetry assumptions (7 to 11) the extended carousel model has 5 new parameters to estimate:

- RGS_{tot}, the total amount RGS protein. The experimental estimations for the abundance of $\mathrm{Sst2^{RGS}}$ in unstimulated cells ranges from 2000 molecules/cell in [22], to 6000 molecules/cell in [17]. Furthermore, this protein is induced during the response [22], something that our model can not capture in its current form. We decided to use the value of 6000 molecules/cell, roughly 3 times the value used for G_{tot} (2045 ± 107 molecules/cell [39]), as this is consistent with our estimation based on YFP tagged proteins for $\mathrm{Sst2^{RGS}}$ and $\mathrm{Ste4^{G\beta}}$ (table S5 and Figure S1).

- V_{cyt}, the cytoplasmic volume accessible to free RGS. As before (section 3.4) we estimated this volume to be 36.4 fl [44].

- $K_d^{R\cdot\mathsf{RGS}}$, the dissociation constant between the RGS and the receptor. To estimate this affinity we noted that in unstimulated cells $\mathrm{Sst2^{RGS}}$ appears to be mainly in the cytoplasm [4]. To assign a numeric value to $K_d^{R\cdot\mathsf{RGS}}$, we solved the amount of RGS in complex with receptors in steady state, which is given by equation 53.

$$\underset{\mathsf{RGS}}{xRy_{ss}} = \frac{1}{2}\left(K_d^{R\cdot\mathsf{RGS}}V_{cyt} + R_{tot} + \mathsf{RGS}_{tot} - \sqrt{(K_d^{R\cdot\mathsf{RGS}}V_{cyt} + R_{tot} + \mathsf{RGS}_{tot})^2 - 4R_{tot}\mathsf{RGS}_{tot})}\right) \tag{53}$$

Here $\underset{\mathsf{RGS}}{xRy_{ss}}$ represents the sum of all species of receptors with RGS at steady state. Using the estimated values for R_{tot}, RGS_{tot} and V_{cyt}, and assuming that only 20%

of Sst2^{RGS} is bound to Ste2^{GPCR} in unstimulated cells ($\underset{\mathsf{RGS}}{xRy_{ss}} = 0.2 \times \mathsf{RGS}_{tot}$), we estimated a value of $K_d^{R\cdot\mathsf{RGS}} = 383\,\text{nM}$.

- $k_{off}^{R\cdot\mathsf{RGS}}$, the *off*-rate between the RGS and the receptor. To our knowledge there is no experimental estimation of this value. We reasoned that if the *off*-rate between receptor and RGS is much faster than the *off*-rate between receptor and G protein ($k_{off}^{R\cdot\mathsf{RGS}} \gg k_{off}^{R\cdot G}$), then the time averaged effect of the RGS would be homogeneous among receptors. Therefore, we used the value $k_{off}^{R\cdot\mathsf{RGS}} = 3\,s^{-1}$ (roughly $30 \times k_{off}^{R\cdot G}$).

- $k_{Hf}^{\overset{xRGt}{\mathsf{RGS}}}$, the hydrolysis rate of Gα^{GTP} bound to receptor with RGS. Based on equation 53 and the estimated values of R_{tot}, RGS_{tot}, V_{cyt} and $K_d^{R\cdot\mathsf{RGS}}$, we estimate that only 37% of the receptors are bound to RGS at any given instant. Equivalently, by ergodicity, a given receptor is bound to RGS only 37% of the time. Therefore, to maintain the same "effective" time-averaged GAP activity used in the simplified carousel model for *wt* abundances of the components (section 3.3), we have to increase the hydrolysis rate in the receptor-RGS-Gα^{GTP} complex accordingly: $k_{Hf}^{\overset{xRGt}{\mathsf{RGS}}} = 0.11\,s^{-1}/0.37 = 0.3\,s^{-1}$ ($0.11\,s^{-1}$ was the rate reported in [52]). Note that in this extended carousel model, according to equation 52, we use $k_{Hf}^{Gt} = k_{Hf}^{xRGt} = 0.002\,s^{-1}$.

3.6 Restriction analysis of the simplified carousel model

To draw conclusions that do not depend on the precise value of the reference parameters (Table 1 in main text), we analyzed the behavior of the carousel model in a large region of parameter space. We varied each parameter 4 orders of magnitude to each side of its reference value, in logarithmic scale. To explore parameter space efficiently we used Latin Hypercube Sampling (LHS) [31]. For each point p in the LHS sample, we numerically simulated a steady-state DoR curve for different receptor abundances, ranging from 30 to 3×10^5 receptors per cell. For each DoR curve we fitted a Hill function (54), and saved the parameters of these fitted curves, namely *Basal*, *Amplitude*, EC_{50} and n (the Hill coefficient).

$$G\beta\gamma = Basal + Amplitude\,\frac{L^n}{EC_{50}^n + L^n} \tag{54}$$

Next, we defined different sets, based on the behavior of the DoR curves. The criteria for each set are defined in equations 55 to 59, where the ^{wt} super index denotes the value of the parameters at WT receptor abundance ($R_{tot} = 3300$ *molecules cell*$^{-1}$) [39], and *max* and *min* are taken over the different receptor abundances. The amount of total G protein was kept constant in the simulations to $G_{tot} = 2040$ *molecules cell*$^{-1}$ [39] and the ligand-receptor dissociation constant was set to one ($K_d^{L\cdot R} = 1$), which is equivalent to adimensionalize the extracellular ligand concentration.

$$S_{wa} = \left\{ p \in LHS \middle| \frac{Basal^{wt}}{G_{tot}} \leq 1/4 \quad \wedge \quad \frac{Amplitude^{wt} + Basal^{wt}}{G_{tot}} \geq 3/4 \right\} \tag{55}$$

$$S_{re} = S_{wa} \cap \left\{ p \in LHS \middle| \frac{max(EC_{50})}{min(EC_{50})} < 10^{0.2} = 1.58 \right\} \tag{56}$$

$$S_{ra} = S_{wa} \cap \left\{ p \in LHS \middle| \frac{max(Amplitude) - min(Amplitude)}{G_{tot}} < 0.2 \right\} \tag{57}$$

$$S_{rr} = S_{re} \cap S_{ra} \tag{58}$$

$$S_{rd} = S_{rr} \cap \left\{ p \in LHS \middle| 0.79 = 10^{-0.1} < \frac{EC_{50}^{wt}}{K_d^{R \cdot L}} < 10^{0.1} = 1.26 \right\} \tag{59}$$

The set S_{wa} (55) corresponds to points in parameter space that have *wt amplitude*; DoR curves with low *Basal* and high *Amplitude* at WT receptor abundance, and therefore are capable of responding to the ligand L. S_{re} (56) are the points with *robust* EC_{50}, defined as the intersection between S_{wa} and the points that maintain a fairly constant EC_{50} at all tested receptor abundances. S_{ra} (57) are the points with *robust Amplitude*, defined as the intersection between S_{wa} and the points that maintain a fairly constant *Amplitude* at all tested receptor abundances. S_{rr} (58) is the intersection between S_{re} and S_{ra}, that is, the points with a *robust response.* These points show robust sensitivity and robust amplitude, and therefore the overall response is independent on variations in receptor abundance. Finally, S_{rd} (59) are the points that show *robust DoR alignment*, and is defined as the intersection between S_{rr} and the points that show a EC_{50} almost equal to the dissociation constant of the receptor $K_D^{R \cdot L}$. These points show both robustness to receptor abundance and *dose-response alignment* (DoRA).

To visualize the distribution of the points in each set, we plotted a matrix of 2D histograms, as shown in Figure S3 for the points in S_{wa}. The panels on the diagonal show the histogram, for the parameters indicated in the top and right margins. Each panel in the upper left triangle shows the 2D histograms for the points in S_{wa}, projected on the plane defined by the parameters labeled on the top (x) and right (y) margins. The frequency of points in each bin is represented by the yellow-red color scale.

The panels on the bottom right triangle show the difference between the frequency of points in S_{wa} that fall in a bin, and the frequency expected assuming independent distributions for the parameters. This *null hypothesis* distributions (H_0), is constructed by combining the marginal distributions represented by the histograms on the diagonal. The difference between the frequency in S_{wa} and H_0 is represented by the blue-red color scale. To determine which panels show a distributions significantly different from H_0, we performed Monte Carlo simulations ($N = 10^5$) using the Cramér-von Mises statistic ($\omega^2 = \sum(\text{Freq} - H_0)^2$), and determined significance using a Bonferroni corrected α. Panels with significant structure are indicated with an asterisk. Note that correspondent panels in the upper an lower triangles of the matrix are labeled with the same number on the top left corner of the panel, and that all parameters are plotted in log_{10} scale.

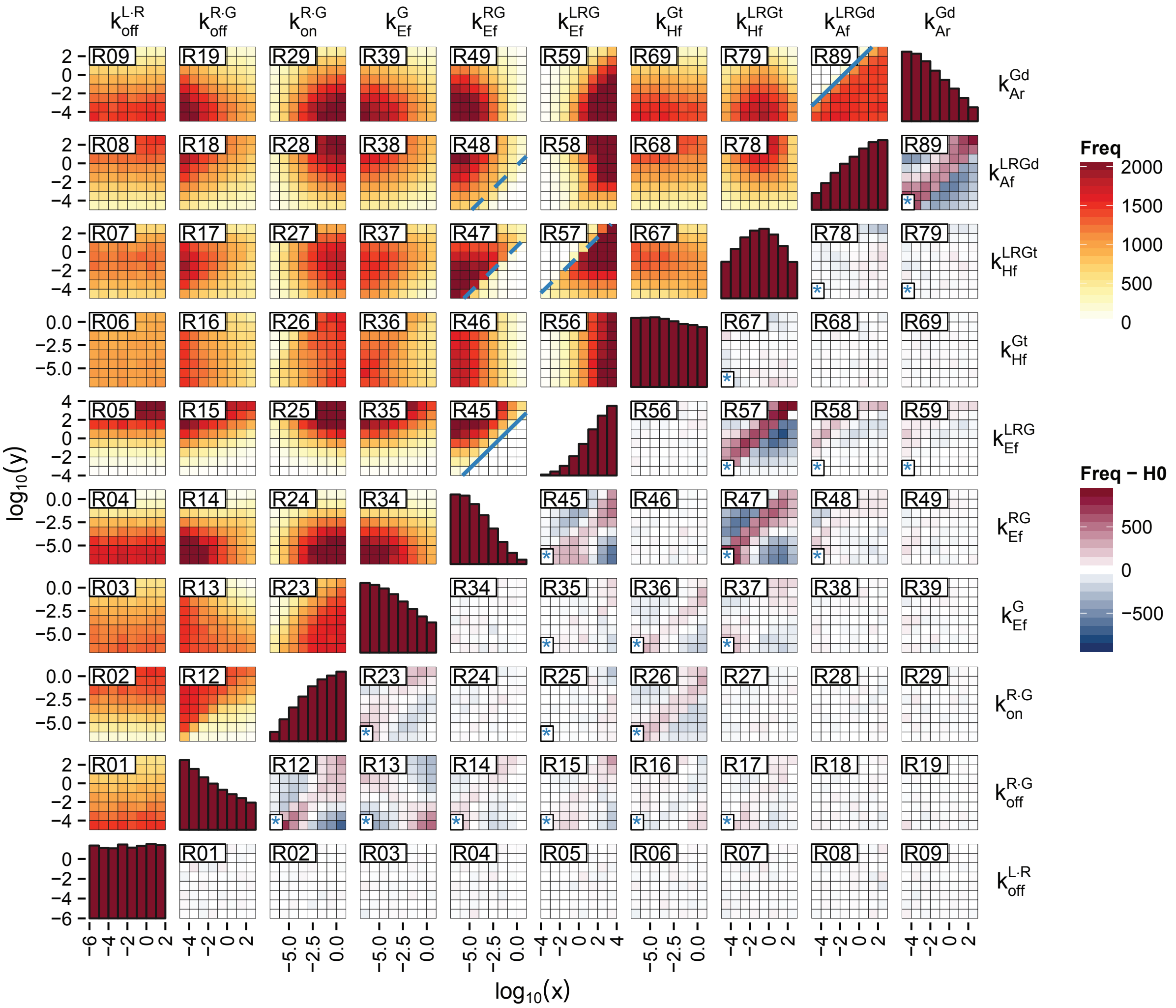

Figure S3. Matrix of 2D histograms showing points in S_{wa}

Many panels of Figure S3 show significant structure (see Table S7 for details). In some of these panels, there are regions almost or totally devoid of points, as in panels R45, R47, R48, R57 and R89. In these cases we say there is a *restriction* between the two parameters that define the panel. For instance, all points in S_{wa} fall on top of the line defined by $log_{10}(k_{Ef}^{LRG}) = 1.7 + log_{10}(k_{Ef}^{RG})$, as shown in panel R45 of Figure S3. This means that points in S_{wa} satisfy the restriction $k_{Ef}^{RG} \overset{R45}{\ll} k_{Ef}^{LRG}$ [3]. In other words, DoR curves with high amplitude and low basal are obtained only when the GEF activity is much higher in ligand-occupied receptors than in unoccupied receptors, which makes intuitive sense.

**Restriction**		**Definition**
$A \lesssim B$	$\Rightarrow$	$\frac{B}{A} \in (0.1, \infty)$
$A < B$	$\Rightarrow$	$\frac{B}{A} \in (1, \infty)$
$A \ll B$	$\Rightarrow$	$\frac{B}{A} \in (10, \infty)$
$A \lll B$	$\Rightarrow$	$\frac{B}{A} \in (100, \infty)$

Table S6. Inequality symbols used to define restrictions. In all cases $A > 0$ and $B > 0$.

Another restriction can be observed in panel R89 of Figure S3, which involves parameters k_{Af}^{LRGd} [4] and k_{Ar}^{Gd}. These two parameters have different units, $s^{-1}nM^{-1}$ and s^{-1} respectively. In order to make them comparable, we express the restriction in terms of G_{tot}, the total number of G proteins, kept fixed in the simulations. Doing this we obtain $K_d^{Gd \cdot G\beta\gamma} = \frac{k_{Ar}^{Gd}}{k_{Af}^{LRGd}} \overset{R89}{\ll} G_{tot}$, which implies complete re-association of $G\beta\gamma$ with $G\alpha$, when $G\alpha$ is in the GDP state.

Because all points in S_{wa} satisfy these two restrictions, we call them *necessary restrictions* and represent them with solid lines in Figure S3. On the other hand, there are panels in which we can define *soft restrictions*. In these panels there are regions almost, but not totally, devoid of points (*e.g.* R47, R48 and R57 in Figure S3). We define soft restrictions such that 95% of the points satisfy them, and represent them with dashed lines.

There are two soft restrictions on k_{Hf}^{LRGt} [5] shown in panels R47 and R57 of Figure S3; $k_{Ef}^{RG} \overset{R47}{<} k_{Hf}^{LRGt} \overset{R57}{<} k_{Ef}^{LRG}$. In other words, the GTP hydrolysis rate has to be slower than the GEF activity of occupied receptor, but higher than the GEF rate of unoccupied receptor. Combining R47 and R57 we get $k_{Ef}^{RG} < k_{Ef}^{LRG}$, which is in line with R45 ($k_{Ef}^{RG} \overset{R45}{\ll} k_{Ef}^{LRG}$). Consistent with this, for $p \in LHS$, $P(R45|R47 \wedge R57) = 0.99$, which means that of the points that satisfy both R47 and R57, 99% also satisfy R45. This kind of relationships between restrictions are shown in Table S8. k_{Hf}^{LRGt} is the only parameter that has upper and lower restrictions for points in S_{wa}, and this is reflected in the 1D histogram for this parameter, which is the only one that shows a maximum at intermediate values.

[3] the precise meaning of $\ll$ is defined in Table S6

[4] under the symmetry assumptions $k_{Af}^{LRGd} = k_{Af}^{RGd} = k_{Af}^{Gd}$

[5] under the symmetry assumptions $k_{Hf}^{LRGt} = k_{Hf}^{RGt}$

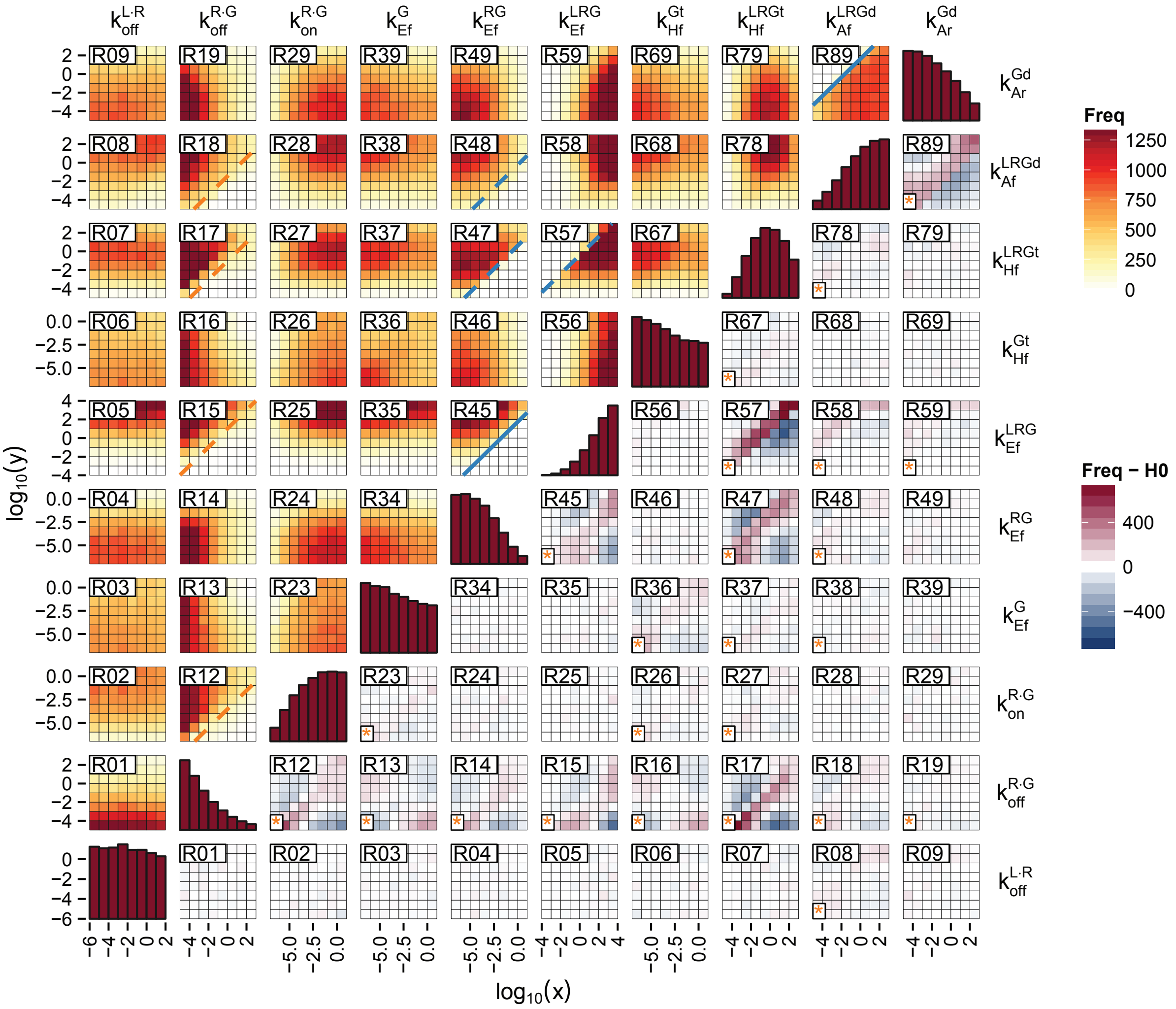

Figure S4. Matrix of 2D histograms showing points in S_{re}

In Figure S4 we show the matrix of 2D histograms for the points in S_{re}, that is, the points that show robust EC_{50} and respond to ligand at wt receptor abundance (belong to S_{wa}). The restrictions defined in Figure S3 are satisfied for points in S_{re} (blue lines in Figure S4), as expected by the fact that $S_{re} \subset S_{wa}$. There are also new restrictions for S_{re}, shown by orange lines in Figure S4, all of which involve the parameter $k_{off}^{R\cdot G}$. Restriction R12 can be expressed as $K_d^{R\cdot G} = \frac{k_{off}^{R\cdot G}}{k_{on}^{R\cdot G}} \overset{R12}{<} G_{tot}$, *i.e.* that the dissociation constant for receptor and $G\alpha$ should be lower than the abundance of G protein. In this condition most receptors will be coupled to G proteins.

The other restrictions compare several rates with $k_{off}^{R\cdot G}$, requiring fast reassociation of $G\beta\gamma$ with $G\alpha$ ($k_{off}^{R\cdot G} \overset{R18}{\ll} G_{tot}\ k_{Af}^{LRGd}$), fast hydrolysis of GTP by receptor coupled $G\alpha$ ($k_{off}^{R\cdot G} \overset{R17}{\lesssim} k_{Hf}^{LRGt}$), and fast nucleotide exchange rate for ligand occupied receptor coupled to $G\alpha$ ($k_{off}^{R\cdot G} \overset{R15}{\ll} k_{Ef}^{LRG}$). Note that all these are soft restrictions, as for each there are 5%

of points in S_{re} that do not satisfy them.

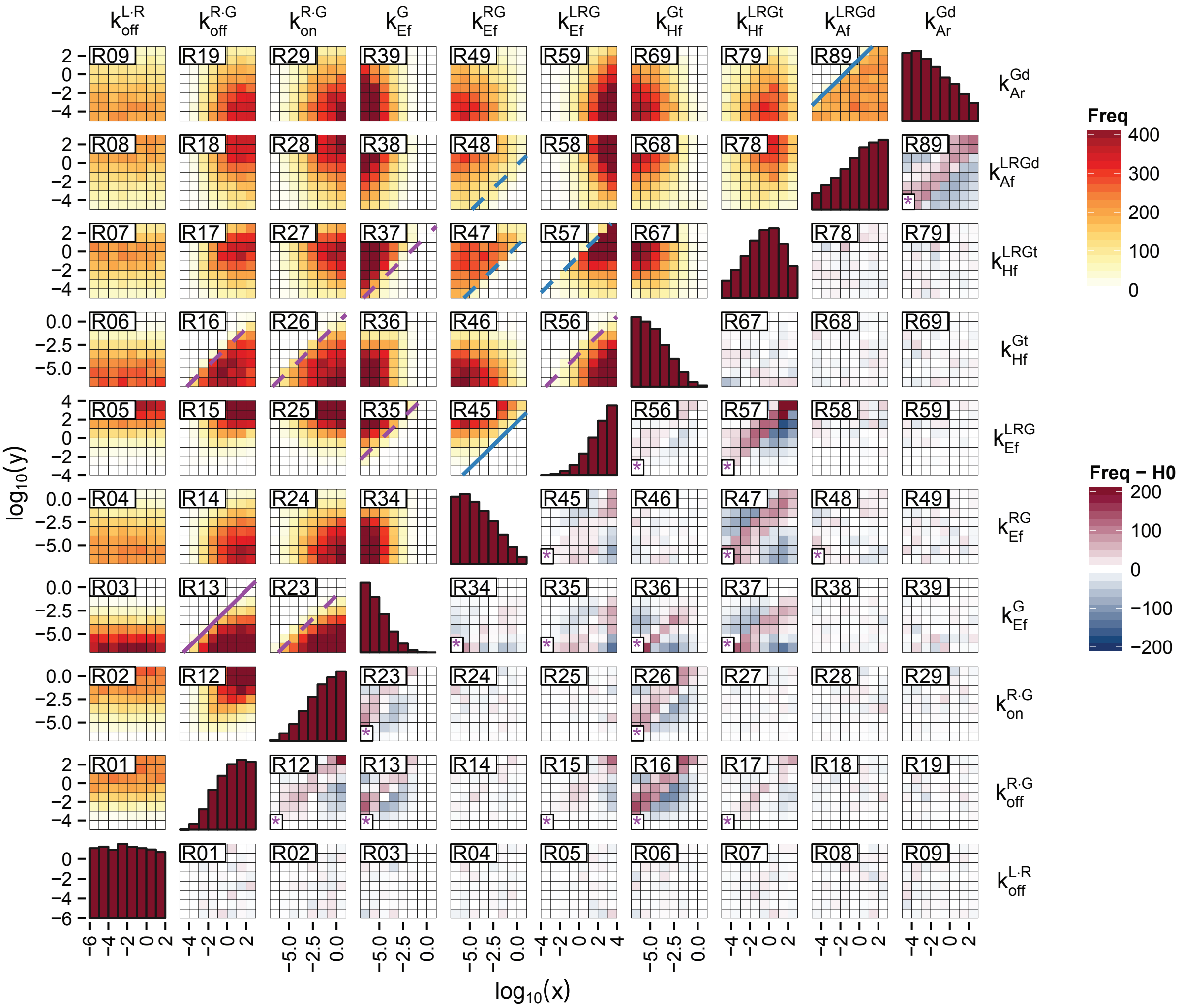

Figure S5. Matrix of 2D histograms showing points in S_{ra}

In Figure S5 we show the matrix of 2D histograms for the points with robust amplitude, that is for S_{ra}. As before, the restrictions for S_{wa} are satisfied, as expected based on the relation $S_{ra} \subset S_{wa}$. There are more restrictions for S_{ra} than for S_{re}, which is consistent with fact that there are four times more points in S_{re} than in S_{ra} (Table S7). The new restrictions are shown in violet in Figure S5. These restrictions involve several parameters, but can be summarized as low k_{Ef}^{G} (R13, R23, R35, R37, Table S7) and low k_{Hf}^{Gt} (R16, R26, R56, Table S7). In line with this, the only new necessary restriction is $k_{off}^{R \cdot G} \overset{R13}{\gg} k_{Ef}^{G}$, *i.e.* that the nucleotide exchange of uncoupled $G\alpha$ has to take much longer than the life time of the $R \cdot G$ complex. In other words, to maintain a robust amplitude, the probability of uncoupled $G\alpha$ to change state has to be low.

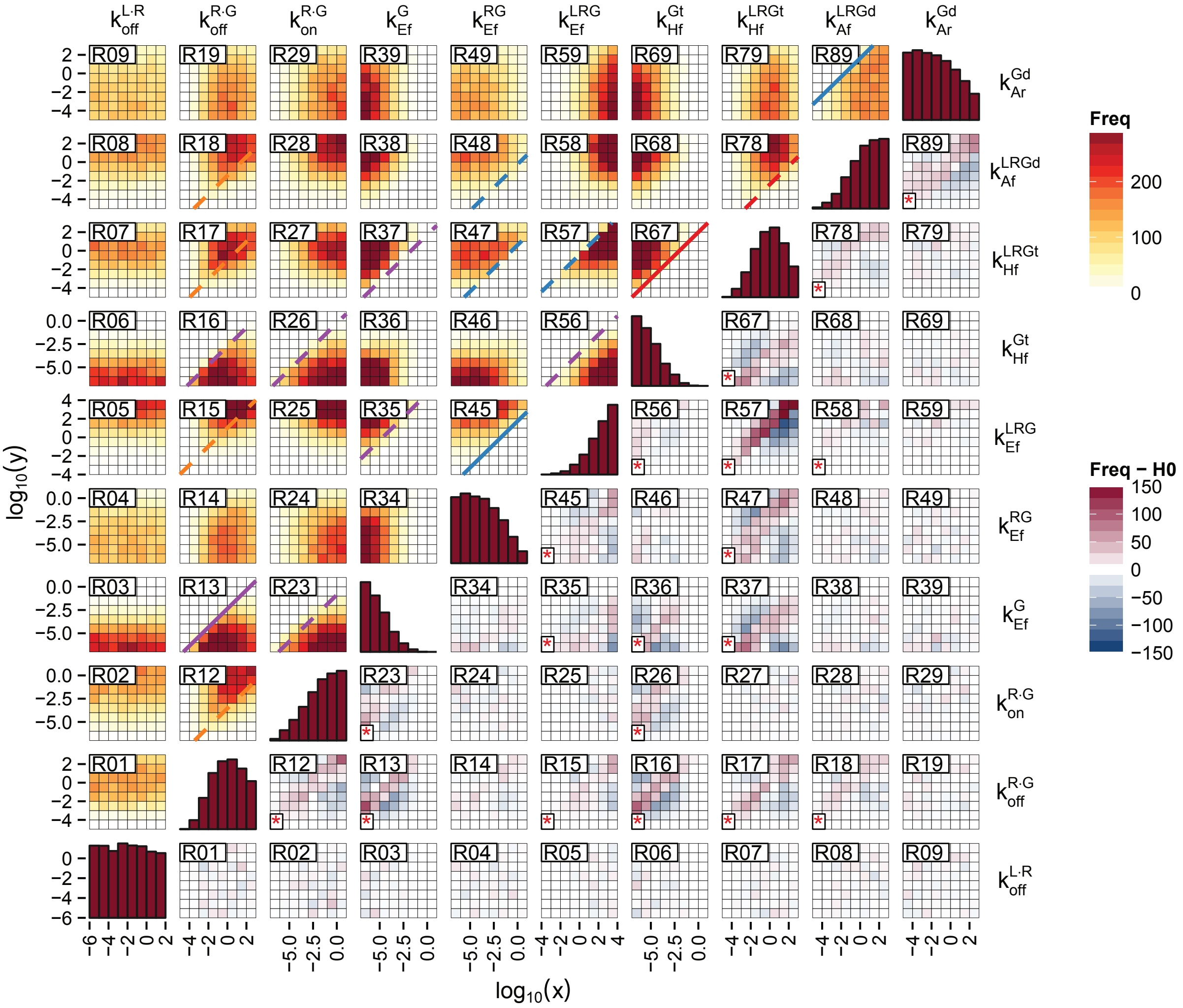

Figure S6. Matrix of 2D histograms showing points in S_{rr}

In Figure S6 we show the points in $S_{rr} = S_{re} \cap S_{ra}$, that is, the points that have robust amplitude and robust EC_{50}, and therefore show a robust overall response. We include in Figure S6 the restrictions defined for S_{wa}, S_{re}, S_{ra} and the new restrictions that appear for S_{rr} (red lines). It is interesting to note that the restrictions for S_{wa} and S_{ra} are satisfied, but the restrictions for S_{re} are not (see R18, R17, R15 and R12 in Figure S6 and in Table S7). These were soft restrictions, therefore the 5% that did not satisfied them in S_{re} are over-represented in S_{rr}. This means that low $k_{off}^{R \cdot G}$ (which results in pre-coupling) is not necessarily required to have a robust response.

There are only two new restrictions for S_{rr} that do not appear in the previous sets. One is that $k_{Hf}^{LRGt} \overset{R78}{<} G_{tot}\, k_{Af}^{LRGd}$, which means that the hydrolysis has to be the rate limiting step in the reassociation of the G protein.

The other new restriction ($k_{Hf}^{Gt} \overset{R67}{\ll} k_{Hf}^{LRGt}$) is a necessary restriction and requires that the hydrolysis rate of uncoupled $G\alpha$ should be much slower than the hydrolysis of receptor

coupled $G\alpha$. Note that this is exactly what we expect in systems in which the RGS physically interacts with the receptor, as this will increase the hydrolysis only when $G\alpha$ is coupled to the receptor. Due to this, we call R67 the *localized RGS* restriction.

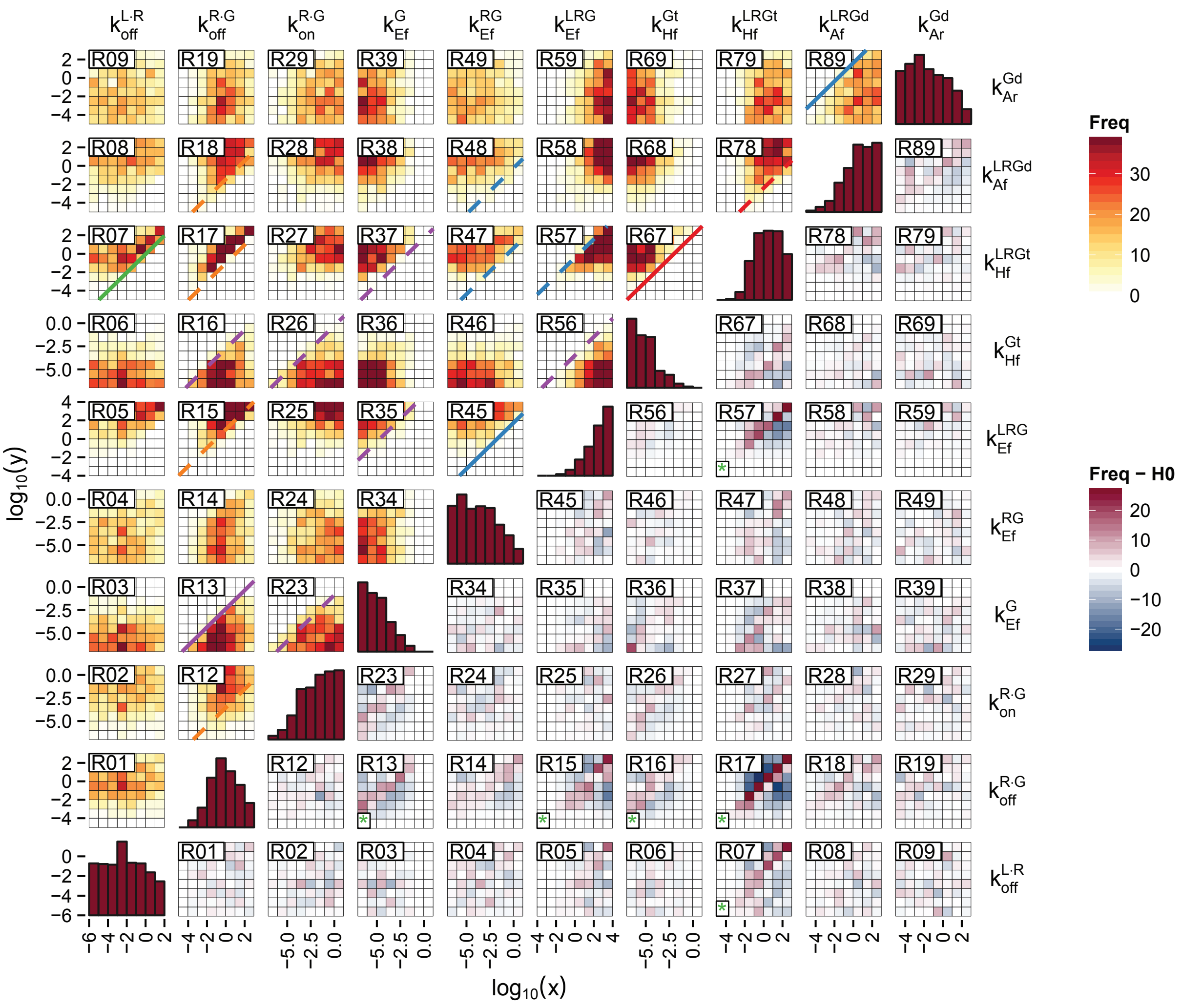

Figure S7. Matrix of 2D histograms showing points in S_{rd}

In Figure S7 we show the points in S_{rd}, that show both a robust response and Dose Response Alignment (DoRA). Interestingly, almost all of the previous restrictions are satisfied for this set, including restrictions for S_{re} like R17. See also Figure 8 and main text for more details.

R[1]	X[2]	[3]	Y[4]	[5]	LHS	S_{wa}	S_{re}	S_{ra}	S_{rr}	S_{rd}
R07	$k_{off}^{L\cdot R}$	$10^{-0.04} <$	k_{Hf}^{LRGt}	$k_{off}^{L\cdot R} < k_{Hf}^{LRGt}$	0.62	0.63	0.72	0.69	0.76	1.00 *
R08	$k_{off}^{L\cdot R}$		k_{Af}^{LRGd}				*			
R12	$k_{off}^{R\cdot G}$	$10^{-3.59} <$	$k_{on}^{R\cdot G}$	$K_d^{R\cdot G} < G_{tot}$	0.67	0.89 *	0.95 *	0.80 *	0.84 *	0.82
R13	$k_{off}^{R\cdot G}$	$10^{-2.40} >$	k_{Ef}^{G}	$k_{off}^{R\cdot G} \ggg k_{Ef}^{G}$	0.46	0.45 *	0.31 *	1.00 *	1.00 *	1.00 *
R14	$k_{off}^{R\cdot G}$		k_{Ef}^{RG}			*	*			
R15	$k_{off}^{R\cdot G}$	$10^{+1.02} <$	k_{Ef}^{LRG}	$k_{off}^{R\cdot G} \ll k_{Ef}^{LRG}$	0.50	0.81 *	0.95 *	0.63 *	0.72 *	0.86 *
R16	$k_{off}^{R\cdot G}$	$10^{-2.70} >$	k_{Hf}^{Gt}	$k_{off}^{R\cdot G} \ggg k_{Hf}^{Gt}$	0.41	0.35 *	0.27 *	0.95 *	0.96 *	0.95 *
R17	$k_{off}^{R\cdot G}$	$10^{-1.03} <$	k_{Hf}^{LRGt}	$k_{off}^{R\cdot G} \lesssim k_{Hf}^{LRGt}$	0.62	0.73 *	0.95 *	0.53 *	0.70 *	1.00 *
R18	$k_{off}^{R\cdot G}$	$10^{-1.46} <$	k_{Af}^{LRGd}	$k_{off}^{R\cdot G} \ll G_{tot}\, k_{Af}^{LRGd}$	0.67	0.83	0.95 *	0.66	0.82	0.89
R19	$k_{off}^{R\cdot G}$		k_{Ar}^{Gd}				*			
R23	$k_{on}^{R\cdot G}$	$10^{-0.87} >$	k_{Ef}^{G}	$k_{on}^{R\cdot G}\, G_{tot} \ggg k_{Ef}^{G}$	0.40	0.61 *	0.53 *	0.95 *	0.93 *	0.88
R25	$k_{on}^{R\cdot G}$		k_{Ef}^{LRG}			*				
R26	$k_{on}^{R\cdot G}$	$10^{-0.26} >$	k_{Hf}^{Gt}	$k_{on}^{R\cdot G}\, G_{tot} \ggg k_{Hf}^{Gt}$	0.47	0.60 *	0.61 *	0.95 *	0.98 *	0.96
R27	$k_{on}^{R\cdot G}$		k_{Hf}^{LRGt}				*			
R34	k_{Ef}^{G}		k_{Ef}^{RG}				*			
R35	k_{Ef}^{G}	$10^{+4.70} <$	k_{Ef}^{LRG}	$k_{Ef}^{G} \lll k_{Ef}^{LRG}$	0.31	0.62 *	0.60	0.95 *	0.95 *	0.96
R36	k_{Ef}^{G}		k_{Hf}^{Gt}			*	*	*	*	
R37	k_{Ef}^{G}	$10^{+1.70} <$	k_{Hf}^{LRGt}	$k_{Ef}^{G} \ll k_{Hf}^{LRGt}$	0.54	0.62 *	0.68 *	0.95 *	1.00 *	1.00
R38	k_{Ef}^{G}		k_{Af}^{LRGd}				*			
R45	k_{Ef}^{RG}	$10^{+1.70} <$	k_{Ef}^{LRG}	$k_{Ef}^{RG} \ll k_{Ef}^{LRG}$	0.64	1.00 *	1.00 *	1.00 *	1.00 *	1.00
R47	k_{Ef}^{RG}	$10^{+0.61} <$	k_{Hf}^{LRGt}	$k_{Ef}^{RG} < k_{Hf}^{LRGt}$	0.66	0.95 *	0.99 *	0.98 *	0.99 *	0.99
R48	k_{Ef}^{RG}	$10^{-0.07} <$	k_{Af}^{LRGd}	$k_{Ef}^{RG} \lll G_{tot}\, k_{Af}^{LRGd}$	0.74	0.95 *	0.96 *	0.94 *	0.97	0.97
R56	k_{Ef}^{LRG}	$10^{-3.50} >$	k_{Hf}^{Gt}	$k_{Ef}^{LRG} \ggg k_{Hf}^{Gt}$	0.44	0.69	0.75	0.95 *	0.99 *	0.99
R57	k_{Ef}^{LRG}	$10^{-0.42} >$	k_{Hf}^{LRGt}	$k_{Ef}^{LRG} > k_{Hf}^{LRGt}$	0.57	0.95 *	0.95 *	0.99 *	1.00 *	1.00 *
R58	k_{Ef}^{LRG}		k_{Af}^{LRGd}			*	*	*		
R59	k_{Ef}^{LRG}		k_{Ar}^{Gd}			*	*			
R67	k_{Hf}^{Gt}	$10^{+2.00} <$	k_{Hf}^{LRGt}	$k_{Hf}^{Gt} \ll k_{Hf}^{LRGt}$	0.50	0.52 *	0.66 *	0.79	1.00 *	1.00
R78	k_{Hf}^{LRGt}	$10^{-2.40} <$	k_{Af}^{LRGd}	$k_{Hf}^{LRGt} < G_{tot}\, k_{Af}^{LRGd}$	0.76	0.85 *	0.86 *	0.86	0.95 *	0.95
R79	k_{Hf}^{LRGt}		k_{Ar}^{Gd}			*				
R89	k_{Af}^{LRGd}	$10^{+1.60} >$	k_{Ar}^{Gd}	$K_d^{Gd\cdot G\beta\gamma} \ll G_{tot}$	0.68	1.00 *	1.00 *	1.00 *	1.00 *	1.00
				Percentage of LHS:	100%	12%	7.2%	1.8%	1.1%	0.13%

Table S7. Parameters restrictions for the different sets defined in eq. 55 to 59. (1) Restriction label as shown in fig. S3 to S7. (2) X variable in the matrix of 2D histograms. (3) Exact relationship defining the restriction. (4) Y variable in the matrix of 2D histograms. (5) Restriction expressed in terms of the inequality operators defined in Table S6 and $G_{tot} = 2042$ *molecules cell*$^{-1}$. Columns LHS, S_{wa}, S_{re}, S_{ra}, S_{rr}, S_{rd} show the fraction of points in each of these sets that satisfy the restrictions. The asterisk indicates significant difference from the null hypothesis obtained assuming independent distributions of the X and Y parameters, calculated using Monte Carlo simulations on the Cramér-von Mises statistic, and a family-wise error rate of $\alpha = 0.05$ (Bonferroni correction).

$R^{(1)}$	Conditional Probability$^{(2)}$
R15	$P(R15\|R17 \wedge R57) = 0.93$
R18	$P(R18\|R17 \wedge R78) = 0.95$
R23	$P(R23\|R12 \wedge R13) = 0.88$
R26	$P(R26\|R12 \wedge R16) = 0.96$
R35	$P(R35\|R15 \wedge R13) = 0.92$
	$P(R35\|R37 \wedge R57) = 0.80$
R37	$P(R37\|R17 \wedge R13) = 0.99$
R45	$P(R45\|R47 \wedge R57) = 0.99$
R48	$P(R48\|R47 \wedge R78) = 0.97$
R56	$P(R56\|R15 \wedge R16) = 1.00$
	$P(R56\|R57 \wedge R67) = 0.96$
R67	$P(R67\|R16 \wedge R17) = 0.997$

Table S8. Relationships between different restrictions. (1) Restriction label as shown in Table S7. (2) Conditional probability of the restriction, for points in LHS.

References

[1] Isabel D Alves, Zdzislaw Salamon, Eva Varga, Henry I Yamamura, Gordon Tollin, and Victor J Hruby. Direct observation of g-protein binding to the human δ-opioid receptor using plasmon-waveguide resonance spectroscopy. *Journal of Biological Chemistry*, 278(49):48890–48897, 2003.

[2] Isabel D Alves, Gilmar FJ Salgado, Zdzislaw Salamon, Michael F Brown, Gordon Tollin, and Victor J Hruby. Phosphatidylethanolamine enhances rhodopsin photoactivation and transducin binding in a solid supported lipid bilayer as determined using plasmon-waveguide resonance spectroscopy. *Biophysical journal*, 88(1):198–210, 2005.

[3] A. Bajaj, A. Celic, F. X. Ding, F. Naider, J. M. Becker, and M. E. Dumont. A fluorescent alpha-factor analogue exhibits multiple steps on binding to its g protein coupled receptor in yeast. *Biochemistry*, 43(42):13564–78, 2004.

[4] D. R. Ballon, P. L. Flanary, D. P. Gladue, J. B. Konopka, H. G. Dohlman, and J. Thorner. Dep-domain-mediated regulation of gpcr signaling responses. *Cell*, 126(6):1079–93, 2006.

[5] Gabriel Biddlecome, Gloria H. Berstein and Elliott M. Ross. Regulation of phospho-

lipase c-beta1 by g(q) and m1 muscarinic cholinergic receptor. *Journal of Biological Chemistry*, 271(14):7999–8007, 1996.

[6] K J Blumer and J Thorner. Beta and gamma subunits of a yeast guanine nucleotide-binding protein are not essential for membrane association of the alpha subunit but are required for receptor coupling. *Proceedings of the National Academy of Sciences of the United States of America*, 87(11):4363–4367, 1990.

[7] Douglas R. Brandts and Elliott M. Ross. GTPase activity of the stimulatory GTP-binding regulatory protein of adenylate cyclase, Gs. *The Journal of biological chemistry*, 260(1):266–272, 1985.

[8] Alan Bush and Alejandro Colman-Lerner. Quantitative measurement of protein relocalization in live cells. *Biophysical journal*, 104(3):727–736, 2013.

[9] Jieming Chen, Nicholas Sawyer, and Lynne Regan. Protein–protein interactions: general trends in the relationship between binding affinity and interfacial buried surface area. *Protein Science*, 22(4):510–515, 2013.

[10] Thomas W Christianson, Robert S Sikorski, Michael Dante, James H Shero, and Philip Hieter. Multifunctional yeast high-copy-number shuttle vectors. *Gene*, 110(1):119–122, 1992.

[11] Chris D Clark, T Palzkill, and D Botstein. Systematic mutagenesis of the yeast mating pheromone receptor third intracellular loop. *Journal of Biological Chemistry*, 269(12):8831–8841, 1994.

[12] A. Colman-Lerner, A. Gordon, E. Serra, T. Chin, O. Resnekov, D. Endy, C. G. Pesce, and R. Brent. Regulated cell-to-cell variation in a cell-fate decision system. *Nature*, 437(7059):699–706, 2005.

[13] A. DeLean, J. M. Stadel, and R. J. Lefkowitz. A ternary complex model explains the agonist-specific binding properties of the adenylate cyclase-coupled β-adrenergic receptor. *Journal of Biological Chemistry*, 255(15):7108–7117, 1980.

[14] M. Dosil, L. Giot, C. Davis, and J. B. Konopka. Dominant-negative mutations in the g-protein-coupled alpha-factor receptor map to the extracellular ends of the transmembrane segments. *Mol Cell Biol*, 18(10):5981–91, 1998.

[15] M. Dosil, K. A. Schandel, E. Gupta, D. D. Jenness, and J. B. Konopka. The c terminus of the saccharomyces cerevisiae alpha-factor receptor contributes to the formation of preactivation complexes with its cognate g protein. *Mol Cell Biol*, 20(14):5321–9, 2000.

[16] Connie Ying Gao and Jennifer L Pinkham. Tightly regulated, beta-estradiol dose-dependent expression system for yeast. *Biotechniques*, 29(6):1226–1231, 2000.

[17] S. Ghaemmaghami, W. K. Huh, K. Bower, R. W. Howson, A. Belle, N. Dephoure, E. K. O'Shea, and J. S. Weissman. Global analysis of protein expression in yeast. *Nature*, 425(6959):737–41, 2003.

[18] Daniel G Gibson, Lei Young, Ray-Yuan Chuang, J Craig Venter, Clyde a Hutchison, and Hamilton O Smith. Enzymatic assembly of DNA molecules up to several hundred kilobases. *Nature Methods*, 6(5):343–345, 2009.

[19] a L Goldstein and J H McCusker. Three new dominant drug resistance cassettes for gene disruption in Saccharomyces cerevisiae - Goldstein - 1999 - Yeast - Wiley Online Library. *Yeast (Chichester, England)*, 15(14):1541–1553, 1999.

[20] Andrew Gordon, Alejandro Colman-Lerner, Tina E Chin, Kirsten R Benjamin, C Yu Richard, and Roger Brent. Single-cell quantification of molecules and rates using open-source microscope-based cytometry. *Nature methods*, 4(2):175–181, 2007.

[21] Anton Graschopf, Jochen a. Stadler, Maria K. Hoellerer, Sandra Eder, Monika Sieghardt, Sepp D. Kohlwein, and Rudolf J. Schweyen. The Yeast Plasma Membrane Protein Alr1 Controls Mg2+ Homeostasis and is Subject to Mg2+-dependent Control of Its Synthesis and Degradation. *Journal of Biological Chemistry*, 276(19):16216–16222, 2001.

[22] N. Hao, N. Yildirim, Y. Wang, T. C. Elston, and H. G. Dohlman. Regulators of g protein signaling and transient activation of signaling: experimental and computational analysis reveals negative and positive feedback controls on g protein activity. *J Biol Chem*, 278(47):46506–15, 2003.

[23] Peter Hein, Francesca Rochais, Carsten Hoffmann, Sandra Dorsch, Viacheslav O Nikolaev, Stefan Engelhardt, Catherine H Berlot, Martin J Lohse, and Moritz Bünemann. Gs activation is time-limiting in initiating receptor-mediated signaling. *Journal of Biological Chemistry*, 281(44):33345–33351, 2006.

[24] Pierre Hentges, Benoit Van Driessche, Lionel Tafforeau, Jean Vandenhaute, and Antony M Carr. Three novel antibiotic marker cassettes for gene disruption and marker switching in schizosaccharomyces pombe. *Yeast*, 22(13):1013–1019, 2005.

[25] L. Hicke, B. Zanolari, and H. Riezman. Cytoplasmic tail phosphorylation of the alpha-factor receptor is required for its ubiquitination and internalization. *J Cell Biol*, 141(2):349–58, 1998.

[26] T. Higashijima, K. M. Ferguson, Murray D Smigel, and Alfred G Gilman. The effect of gtp and mg2+ on the gtpase activity and the fluorescent properties of g(o), 1987.

[27] Duane D Jenness, Anne C Burkholder, and Leland H Hartwell. Binding of a-factor Pheromone to Yeast a Cells: Chemical and Genetic Evidence for an a-Factor Receptor. *Genetic Analysis*, 35(December):521–529, 1983.

[28] J.B. Konopka, D.D. Jenness, and L.H. Hartwell. The c-terminus of the s. cerevisiae alpha-pheromone receptor mediates an adaptive response to pheromone. *Cell*, 54(5):609–620, 1988.

[29] L. M. Leavitt, C. R. Macaluso, K. S. Kim, N. P. Martin, and M. E. Dumont. Dominant negative mutations in the alpha-factor receptor, a g protein-coupled receptor encoded by the ste2 gene of the yeast saccharomyces cerevisiae. *Mol Gen Genet*, 261(6):917–32, 1999.

[30] Mark S. Longtine, Amos McKenzie, Douglas J. Demarini, Nirav G. Shah, Achim Wach, Arndt Brachat, Peter Philippsen, and John R. Pringle. Additional modules for versatile and economical PCR-based gene deletion and modification in Saccharomyces cerevisiae. *Yeast*, 14(10):953–961, 1998.

[31] Michael D McKay, Richard J Beckman, and William J Conover. Comparison of three methods for selecting values of input variables in the analysis of output from a computer code. *Technometrics*, 21(2):239–245, 1979.

[32] Suchetana Mukhopadhyay and Elliott M Ross. Rapid gtp binding and hydrolysis by gq promoted by receptor and gtpase-activating proteins. *Proceedings of the National Academy of Sciences*, 96(17):9539–9544, 1999.

[33] Mark C Overton, Sharon L Chinault, and Kendall J Blumer. Oligomerization of g-protein-coupled receptors: lessons from the yeast saccharomyces cerevisiae. *Eukaryotic cell*, 4(12):1963–1970, 2005.

[34] P. M. Pryciak and F. A. Huntress. Membrane recruitment of the kinase cascade scaffold protein ste5 by the gbetagamma complex underlies activation of the yeast pheromone response pathway. *Genes Dev*, 12(17):2684–97, 1998.

[35] J. E. Reneke, K. J. Blumer, W. E. Courchesne, and J. Thorner. The carboxy-terminal segment of the yeast alpha-factor receptor is a regulatory domain. *Cell*, 55(2):221–34, 1988.

[36] A. Shah and L. Marsh. Role of sst2 in modulating g protein-coupled receptor signaling. *Biochemical and biophysical research communications*, 226(1):242–246, 1996.

[37] Robert S Sikorski and Philip Hieter. A system of shuttle vectors and yeast host strains designed for efficient manipulation of dna in saccharomyces cerevisiae. *Genetics*, 122(1):19–27, 1989.

[38] Jennifer Terrell, Susan Shih, Rebecca Dunn, and Linda Hicke. A function for monoubiquitination in the internalization of a g protein–coupled receptor. *Molecular cell*, 1(2):193–202, 1998.

[39] T. M. Thomson, K. R. Benjamin, A. Bush, T. Love, D. Pincus, O. Resnekov, R. C. Yu, A. Gordon, A. Colman-Lerner, D. Endy, and R. Brent. Scaffold number in yeast signaling system sets tradeoff between system output and dynamic range. *Proc Natl Acad Sci U S A*, 108(50):20265–70, 2011.

[40] Ty Thomson. Yeast Pheromone Model, 2007.

[41] Ty M Thomson. *Models and Analysis of Yeast Mating Response: Tools for Model Building, From Documentation to Time-Dependent Stimulation.* PhD thesis, Massachusetts Institute of Technology, 2008.

[42] Junko Y Toshima, Jiro Toshima, Marko Kaksonen, Adam C Martin, David S King, and David G Drubin. Spatial dynamics of receptor-mediated endocytic trafficking in budding yeast revealed by using fluorescent α-factor derivatives. *Proceedings of the National Academy of Sciences*, 103(15):5793–5798, 2006.

[43] Thomas W Traut. Physiological concentrations of purines and pyrimidines. *Molecular and cellular biochemistry*, 140(1):1–22, 1994.

[44] Maho Uchida, Yidi Sun, Gerry McDermott, Christian Knoechel, Mark A Le Gros, Dilworth Parkinson, David G Drubin, and Carolyn A Larabell. Quantitative analysis of yeast internal architecture using soft x-ray tomography. *Yeast*, 28(3):227–236, 2011.

[45] F. van Drogen, V. M. Stucke, G. Jorritsma, and M. Peter. Map kinase dynamics in response to pheromones in budding yeast. *Nat Cell Biol*, 3(12):1051–9, 2001.

[46] Alejandra C Ventura, Alan Bush, Gustavo Vasen, Matías A Goldín, Brianne Burkinshaw, Nirveek Bhattacharjee, Albert Folch, Roger Brent, Ariel Chernomoretz, and Alejandro Colman-Lerner. Utilization of extracellular information before ligand-receptor binding reaches equilibrium expands and shifts the input dynamic range. *Proceedings of the National Academy of Sciences*, 111(37):E3860–E3869, 2014.

[47] Achim Wach. Pcr-synthesis of marker cassettes with long flanking homology regions for gene disruptions in s. cerevisiae. *Yeast*, 12(3):259–265, 1996.

[48] J. L. Weiner, C. Guttierez-Steil, and K. J. Blumer. Disruption of receptor-G protein coupling in yeast promotes the function of an SST2-dependent adaptation pathway. *Journal of Biological Chemistry*, 268(11):8070–8077, 1993.

[49] J M Weiss, P H Morgan, M W Lutz, and T P Kenakin. The cubic ternary complex receptor-occupancy model i. model description. *Journal of theoretical biology*, 178(4):151–167, 1996.

[50] Yuh-Lin Wu, Shelley B Hooks, T Kendall Harden, and Henrik G Dohlman. Dominant-negative inhibition of pheromone receptor signaling by a single point mutation in the G protein alpha subunit. *The Journal of biological chemistry*, 279(34):35287–97, aug 2004.

[51] J Wyman. The turning wheel: a study in steady states. *Proceedings of the National Academy of Sciences of the United States of America*, 72(10):3983–3987, 1975.

[52] T. M. Yi, H. Kitano, and M. I. Simon. A quantitative characterization of the yeast heterotrimeric g protein cycle. *Proc Natl Acad Sci U S A*, 100(19):10764–9, 2003.
