# Supplementary material for: Yeast GPCR signaling reflects the fraction of occupied receptors, not the number
Source: Mol Syst Biol. 2016 Dec 29;12(12):898. doi: 10.15252/msb.20166910 (PMC5199120; doi:10.15252/msb.20166910)
Supplement: Supplementary file 2 — Expanded View Figures PDF [file MSB-12-898-s002.pdf]

Expanded View Figures

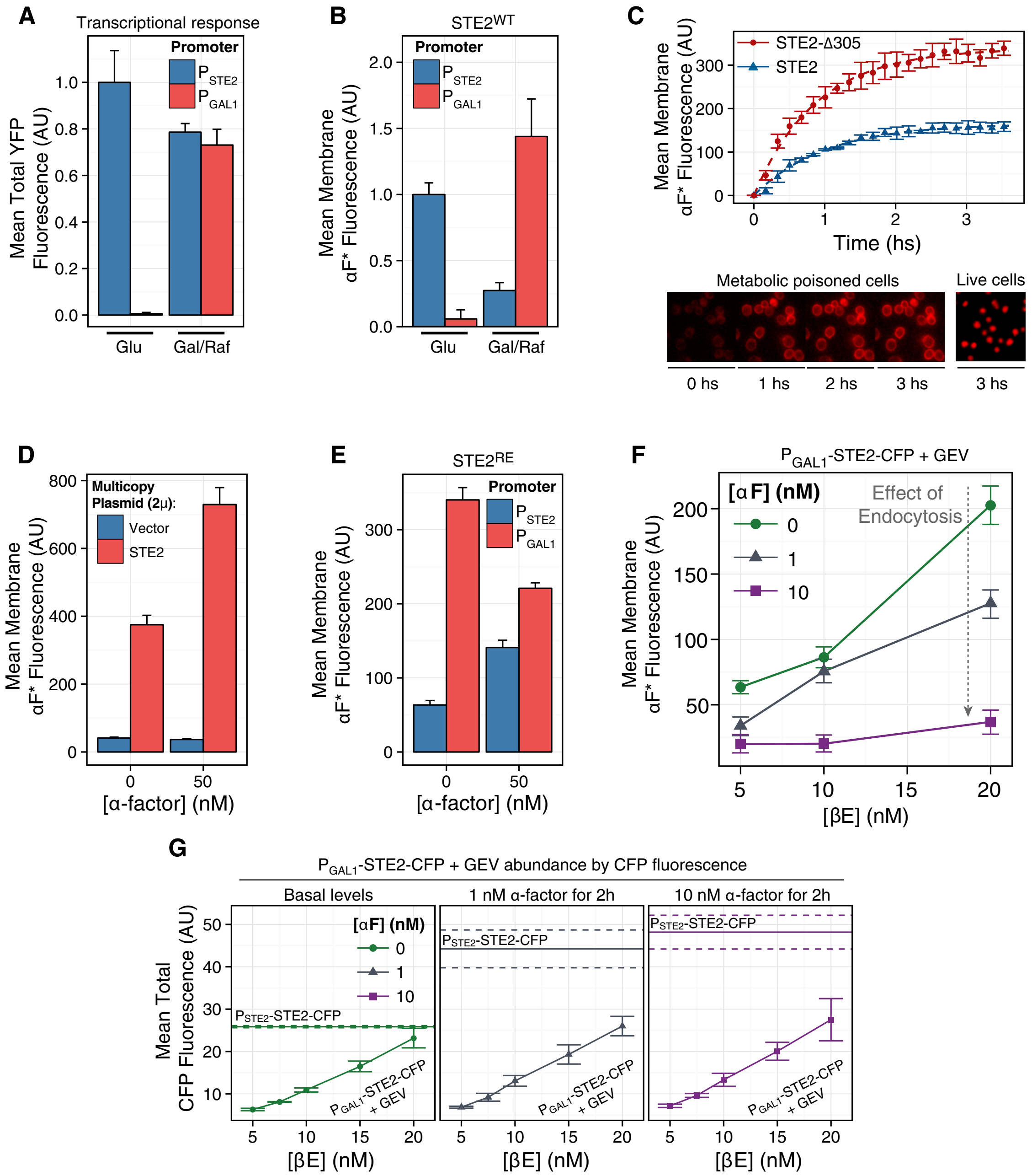

Figure EV1.

Figure EV1. Abundance of Ste2^{GPCR} at the plasma membrane in the used strains.

A P_{GAL1}-*STE2* strains do not induce a PRS transcriptional reporter in glucose. We stimulated P_{STE2}-*STE2* (TCY3154, blue bars) or P_{GAL1}-*STE2* (YAB5302, red bars) expressing the P_{PRM1}-*YFP* reporter with 1 μM α-factor for 2 h in the indicated media, and then, we measured the accumulated YFP by image cytometry. The autofluorescence of ACL394 was subtracted and the values normalized to the value of TCY3154 in SC-glucose.

B P_{STE2}-*STE2* vs. P_{GAL1}-*STE2* binding sites in Gal/Raff. We measured Ste2 abundance at the plasma membrane in cells from a (see Appendix). Before stimulation, WT cells have 0.3-fold Ste2 abundance relative to WT in SC-glucose, while P_{GAL1}-*STE2* have 1.5-fold. The background level of Δ*ste2* strain (TCY3002) was subtracted and the values normalized to WT TCY3154 in SC-glucose.

C Metabolic poisons block endocytosis. Time course of fluorescent α-factor binding. We added metabolic poisons (NaF 10 mM plus 10 mM sodium azide) to exponentially growing WT *STE2* cells (ACL379, blue triangles) or the endocytosis-deficient mutant C-terminal-tail-truncated *STE2-Δ305* mutant (YAB3724, red circles). Then, we added fluorescent α-factor and imaged cells over time in the fluorescence microscope (see Appendix). Plot shows the mean membrane fluorescence over time of a representative experiment. Error bars correspond to the 95% CI. Images show the increasing peripheral (plasma membrane) staining over time in the metabolically poisoned yeast (left) and the strong signal detected inside the vacuole of living yeast (not incubated with the poisons).

D–G Ste2 abundance in yeast over- or underexpressing Ste2. We cultured ACL394 yeast with the multicopy 2μ plasmids containing or not *STE2* (YEp24-STE2(ACY5553) and pRS426 (ACY5552)) (D), expressing the Ste2^{RE}-CFP reduced endocytosis mutant under the P_{STE2} or P_{GAL1} (YGV5563 and YGV5565) (E), or expressing GEV plus P_{GAL1}-*STE2-CFP* (YIP5581) in SC-glucose (F, G) or SC-Gal/Raff (E), in the presence or absence of the indicated α-factor concentration for two hours. At this time, we measured Ste2 abundance with fluorescent α-factor (D–F) or CFP total fluorescence (G).

Data information: Data correspond to the mean ± SEM (G) or 95% CI (D–F). In (F), we note the strong reducing effect that increasing α-factor had on Ste2 abundance. In contrast, strains with reduced endocytosis (E) do not show this dependence on α-factor. As a reference, in (G) we indicate with a horizontal line (dashed line mark SEM) of the values obtained in WT STE2-CFP strains. Data correspond to three biological replicates. (A–F) Error bars represent the 95% CI. (G) Error bars represent the SEM.

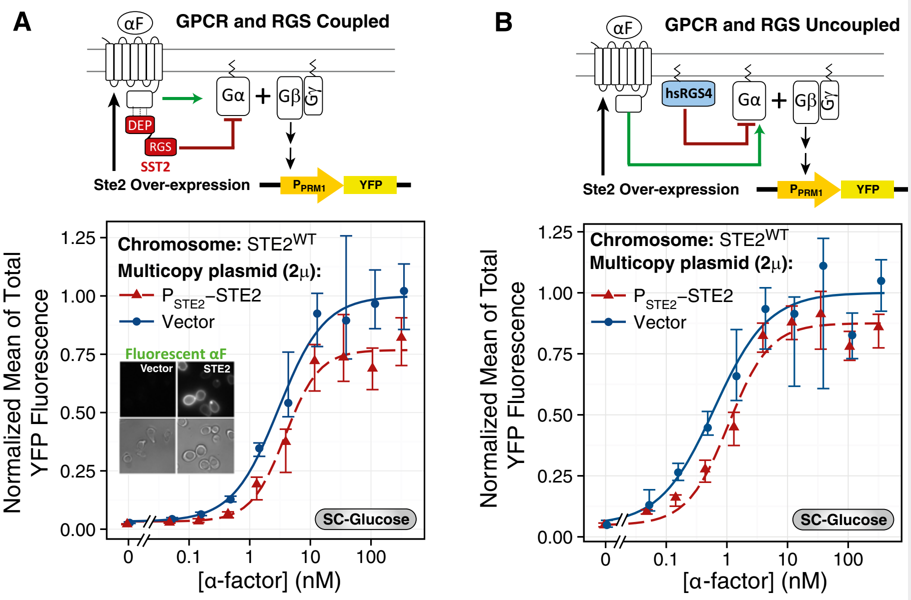

Figure EV2. Transcriptional reporters and Ste2 overexpression. RGS-independent robustness.

A We used strain TCY394 containing the PRS-inducible P_{PRM1}-*YFP* transformed with the multicopy 2μ plasmid containing the *STE2* gene (YEp24-STE2, ACY5552, red triangles) vs. empty vector (pRS426, ACY5553, blue circles), resulting in a 20- to 40-fold difference in Ste2 abundance (as depicted in the inset and shown in Fig EV1D). We grew cells in SC-glucose and then stimulated them with the indicated α-factor concentrations and 10 μM 1NM-PP1 for two hours. We then measured accumulated YFP.

B Same as in (A) but using strains in which we uncoupled the GPCR and the RGS by replacing *SST2* with *hsRGS4*, as in Fig 4. We used strains with 2μ plasmids containing (ACY5649, dashed red) or not (ACY5648, solid blue) the *STE2* gene.

Data information: Data correspond to the mean total YFP, and error bars show the 95% CI calculated by bootstrapping. Note that overexpressing strains show a reduced response relative to WT, independent of the GPCR–RGS interaction, as in Fig 5. Data correspond to a representative experiment of three independent assays.

 © 2016 The Authors

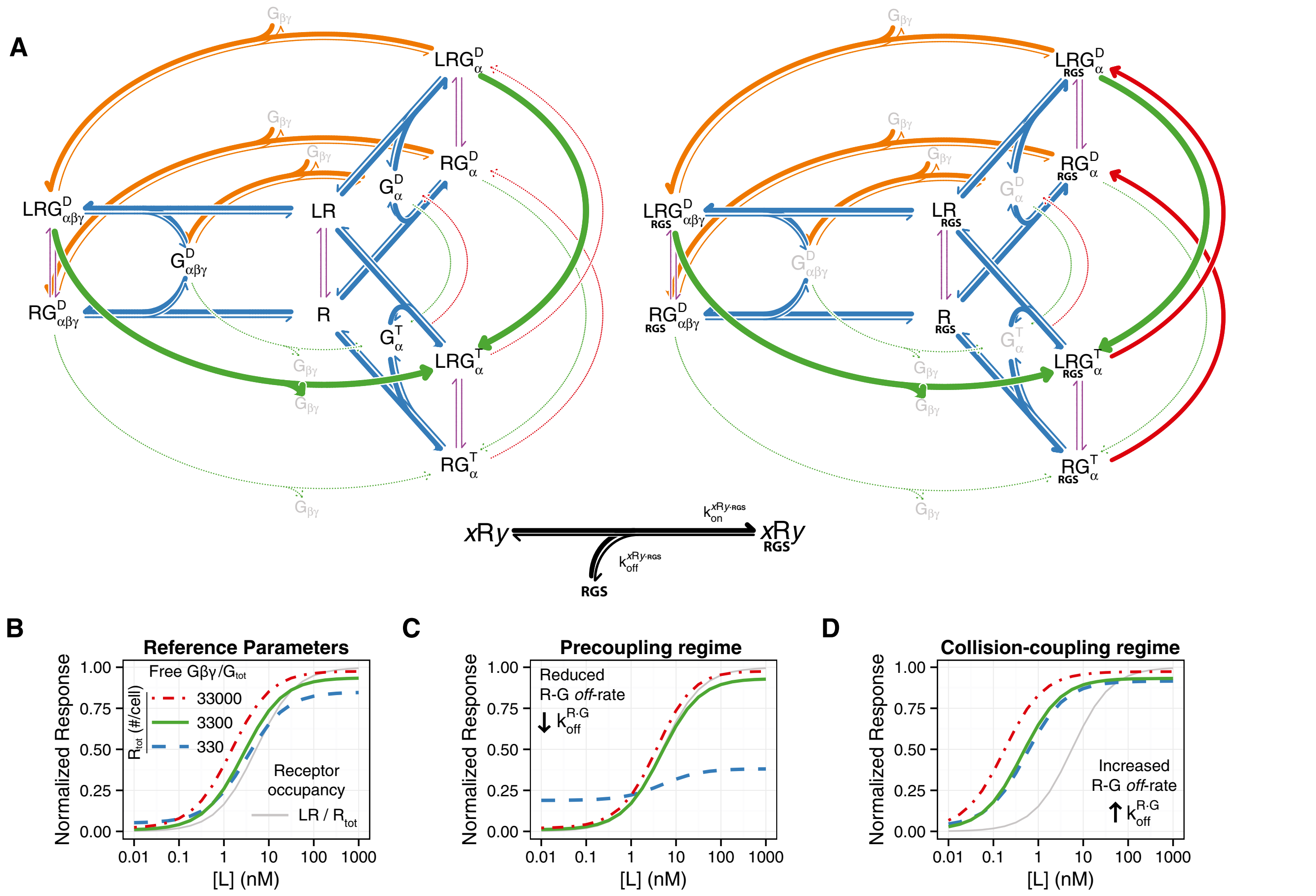

Figure EV3. Extended *carousel* model with RGS interaction.

A Extended *carousel* model scheme, explicitly including the association between the RGS and the receptor. The left part of the scheme represents the transitions between RGS-free receptors, while the right part represents the corresponding transitions of RGS-bound receptor. Duplicated species are colored gray. The black arrows at the bottom represent the RGS–receptor association reaction. In this notation, $x \in \{\emptyset, L\}$ represents the ligand-bound state of the receptor, and $y \in \{\emptyset, G, Gt, Gd\}$ represents the G-protein-coupled state of the receptor (uncoupled or coupled to $G^D_{\alpha\beta\gamma}$, G^T_α, G^D_α, respectively) resulting in eight possible RGS–receptor association reactions. See model definition, symmetry assumptions, and parameter estimation in the Appendix. The parameters are those in Table 1, plus $RGS_{tot} = 6{,}000$ molecules cell^{-1}, $V_{cyt} = 36.4$ fl, $K_d^{R \cdot RGS} = 383$ nM, and $k_{Hf}^{xRRGSGt} = 0.002\ s^{-1}$.

B–D (B) Steady-state dose-response curves, resulting from the extended *carousel* model. The gray curve represents fractional occupancy of the receptor. Color curves show the ratio of free Gβγ to G_{tot}, for different receptor abundances R_{tot}. (C) Same as in (B) for $k_{off}^{R \cdot G} = 0.001\ s^{-1}$. (D) Same as in (B) for $k_{off}^{R \cdot G} = 10\ s^{-1}$.

© 2016 The Authors

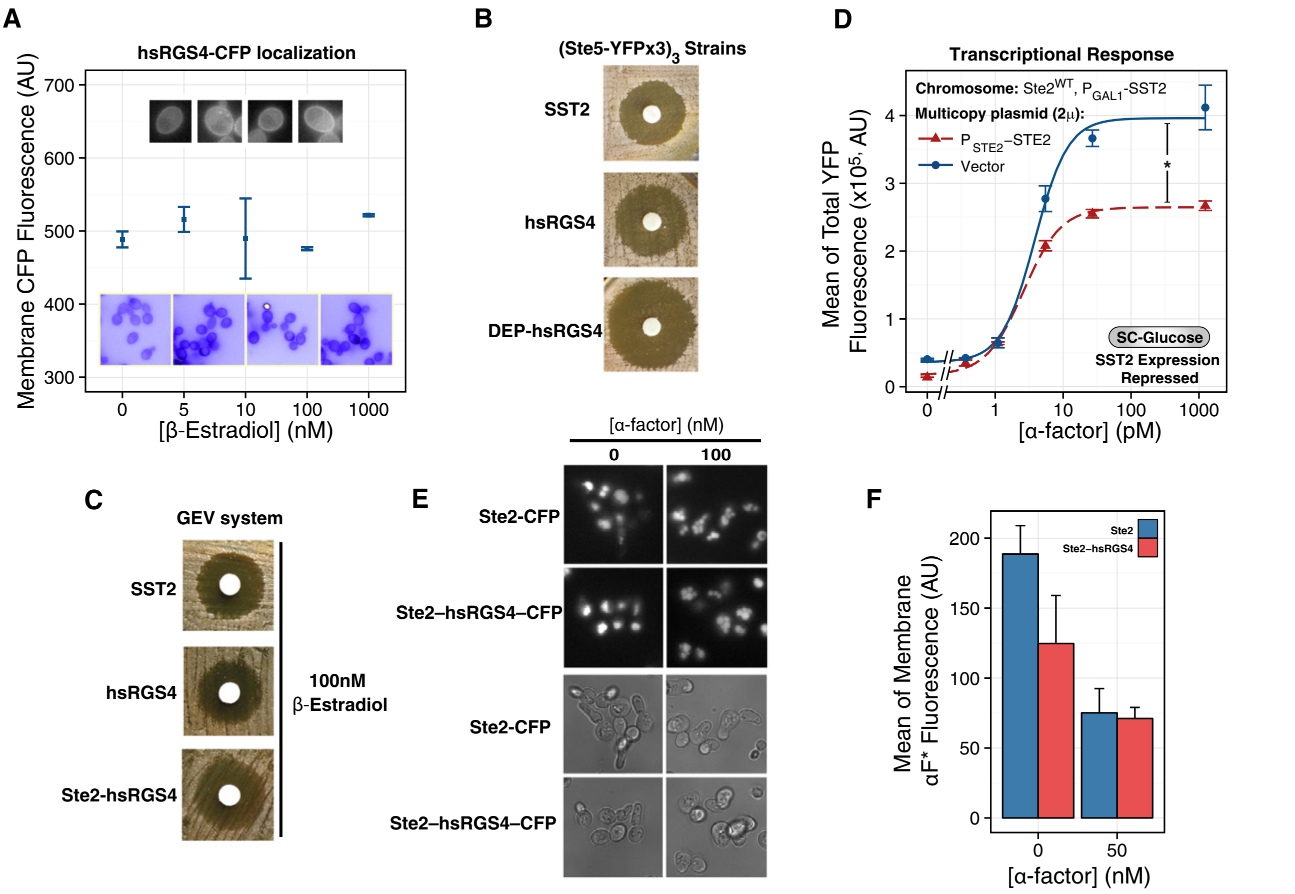

Figure EV4. Characterization of alternate RGS-expressing strains.

A hsRGS4-CFP localizes mainly to the plasma membrane in a manner independent of Ste2. We grew a GEV system strain expressing P_{ACT1}-hsRGS4 (YIP5370) as the single RGS in SC-glucose, then added the indicated β-estradiol concentrations for three hours, and acquired images in the CFP channel. Plot shows average membrane fluorescence. Error bars show the 95% CI of the mean of three biological replicates. Insets show two magnifications of representative yeast.

B, C Sensitivity to pheromone-induced cell cycle arrest of RGS4-expressing strains. We performed halo assays of P_{STE2}-*STE2* (B, YPP3662, ACY5612, and ACY5614) or GEV system P_{GAL1}-*STE2* (C, YIP5581, YGV5642, and YGV5620) strains. Note that the DEP-hsRGS4 strain has a halo larger than WT, indicating enhanced sensitivity to α-factor.

D Overexpression of Ste2 in the absence of Sst2. We grew P_{PRM1}-*YFP* reporter strains with P_{GAL1}-*SST2* under repression conditions (SC-glucose) with 2μ plasmids containing (YGV5653, dashed red) or not (YGV5652, solid blue) the *STE2* gene and added the indicated α-factor concentration for two hours in the presence of 10 μM 1NM-PP1 before measuring accumulated YFP reporter. Plot shows dose responses, and data correspond to the mean ± SEM accumulated YFP from three independent experiments. Note that overexpressing strains show a reduced response relative to WT. For statistical significance, we compared the coefficients obtained from non-linear mixed-effects fit to a Hill-function model. For amplitude, vector: (359,979 ± 12,117) AU vs. 2μ *STE2* (247,639 ± 8,408) AU, $P < 10^{-4}$ (*).

E, F The Ste2-hsRGS4 fusion is endocytosed similar to WT. We grew P_{STE2}-*STE2-CFP* (YGV5560) or P_{STE2}-*STE2-RGS4-CFP* (YGV5678) yeast in SC-glucose and added or not 100 nM (E) or 50 nM (F) α-factor for 2 hours before imaging in the CFP channel (E) or measuring $Ste2^{GPCR}$ plasma membrane abundance with fluorescent α-factor (F). In (E), we show images in the CFP channel (top) or brightfield (bottom). Note the bright signal from inside cells (the vacuole). Bar plot in (F) shows the mode membrane fluorescence for the indicated strains. Error bars show the 95% CI of the mean of three biological replicates.

 © 2016 The Authors

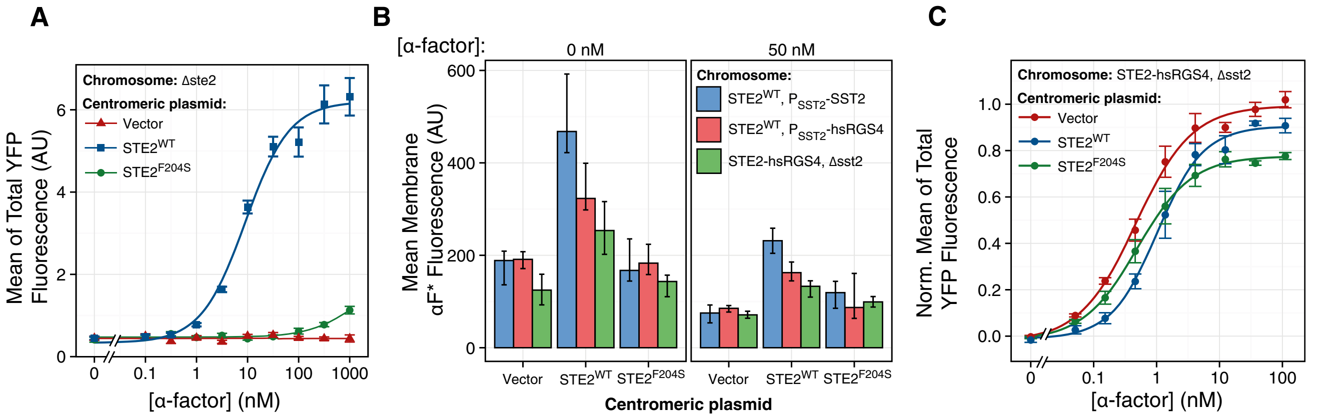

Figure EV5. Characterization of STE2^{GPCR} mutant alleles.

A–C (A, C) We grew P_{PRM1}-*YFP* strain Δ*ste2* (A, YAB4075, YAB4078, YAB4079) or P_{STE2}-*Ste2-hsRGS4-CFP* Δ*sst2* (C, YGV5678-80) transformed with empty plasmid (red triangles), a plasmid with the *STE2* gene (blue squares) or the *STE2^{F204S}* mutant (green circles), stimulated them with the indicated α-factor concentrations and 10 μM 1NM-PP1, and 2 h later imaged to measure accumulated YFP. Data correspond to the mean YFP ± SEM of 3 biological replicates. (B) We grew P_{PRM1}-YFP strains with P_{STE2}-*STE2* P_{SST2}-*SST2* (YGV5666-68), P_{STE2}-*STE2* P_{SST2}-*hsRGS4-CFP* (YGV5669-71), *or* P_{STE2}-*STE2-RGS4-CFP* Δ*sst2* (YGV5678-80) and then stimulated them or not with 50 nM α-factor for two hours. We then measured Ste2 abundance at the plasma membrane using fluorescent α-factor.
